# Wild and farmed salmon (*Salmo salar*) as reservoirs for infectious salmon anaemia virus, and the importance of horizontal- and vertical transmission

**DOI:** 10.1371/journal.pone.0215478

**Published:** 2019-04-16

**Authors:** Are Nylund, Jarle Brattespe, Heidrun Plarre, Martha Kambestad, Marius Karlsen

**Affiliations:** 1 University of Bergen, Fish Diseases Research Group, Bergen, Norway; 2 PHARMAQ AS, Oslo, Norway; Friedrich-Loeffler-Institut Bundesforschungsinstitut fur Tiergesundheit, GERMANY

## Abstract

The infectious salmon anaemia virus (ISAV) is an important pathogen on farmed salmon in Europe. The virus occurs as low- and high virulent variants where the former seem to be a continuous source of new high virulent ISAV. The latter are controlled in Norway by stamping out infected populations while the former are spreading uncontrolled among farmed salmon. Evidence of vertical transmission has been presented, but there is still an ongoing discussion of the importance of circulation of ISAV via salmon brood fish. The only known wild reservoirs are in trout (*Salmo trutta*) and salmon (*Salmo salar*). This study provides the first ISAV sequences from wild salmonids in Norway and evaluates the importance of this reservoir with respect to outbreaks of ISA among farmed salmon. Phylogenetic analyses of the surface protein hemagglutinin-esterase gene from nearly all available ISAV from Norway, Faeroe Islands, Scotland, Chile and wild salmonids in Norway show that they group into four major clades. Including virulent variants in the analysis show that they belong in the same four clades supporting the hypothesis that there is a high frequency of transition from low to high virulent variants in farmed populations of salmon. There is little support for a hypothesis suggesting that the wild salmonids feed the virus into farmed populations. This study give support to earlier studies that have documented local horizontal transmission of high virulent ISAV, but the importance of transition from low- to high virulent variants has been underestimated. Evidence of vertical transmission and long distance spreading of ISAV via movement of embryos and smolt is presented. We recommend that the industry focus on removing the low virulent ISAV from the brood fish and that ISAV-free brood fish salmon are kept in closed containment systems (CCS).

## Introduction

Infectious salmon anaemia virus (ISAV), an orthomyxovirus infecting farmed and wild members of the genus *Salmo* is an economically important pathogen of farmed Atlantic salmon (*Salmo salar*). It is also one of the most studied viruses among those causing mortalities in farmed Atlantic salmon. The disease, infectious salmon anaemia (ISA), was officially discovered in 1984 among salmon parr in a hatchery in Western Norway [1]. The disease resulted in about 80% mortality at the site. In the following years the disease emerged in marine farms along the Norwegian coast that received smolt from this hatchery [2]. The number of outbreaks increased steadily from 1984 until 1991, a year with around 80 outbreaks. The large losses due to ISA triggered changes in the production of Atlantic salmon and the production went from multiple to one single generation at each production site. This lead to a new situation in 1994 with only a single outbreak of ISA [3]. The pathology of ISA was first described in 1991 [4].

The emergence of ISA did also trigger increased research efforts focusing on this disease in Norway. It was shown in 1987 [1] that ISA was a transmissible disease and this study was followed by several transmission experiments all resulting in high mortalities in the challenged groups of salmon [2, 5, 6, 7, 8, 9, 10, 11, 12, 13]. All tissues, mucus and faeces from infected salmon contained the agent causing ISA [8, 10, 12], and in 1992 it was shown that the salmon louse, *Lepeophtheirus salmonis*, could act as a mechanical vector for the agent causing ISA [14]. In the next two years it was also shown that the causative agent of ISA was able to infect and multiply in trout (*Salmo trutta*) that could also transmit the agent to healthy salmon [15, 16, 17, 18, 19]. The ISA virus, virons and target cells, was first detected in challenged salmon in 1993 [9] and the year after in farmed salmon collected from outbreaks of ISA [20, 21, 22]. The virus was cultured in 1995 in a newly developed cell culture, SHK cells [23, 24] and later in an established cell line from Atlantic salmon [25].

In the years after 1994 the number of outbreaks of ISA in Norway increased to about 10 cases every year, and in 1997 the virus emerged in Canada and a year later in Scotland [26, 27, 28, 29, 30]. At present the ISA virus and outbreaks of the disease has also been detected in Shetland, USA, Faeroe Islands, and Chile [31, 32, 33, 34, 35, 36]. The ISA viruses found outside Norway, with the exception of viruses from Chile, were genetically distinct from the Norwegian ISA viruses. The emergences of the ISA virus outside Norway lead an increased effort to gain knowledge about reservoirs and transmission routes for the virus. After several challenge experiments of different fish species and screening of fresh water and marine fish species for presence of the ISA virus the only reservoir species seem to be salmonids with *S*. *salar* and *S*. *trutta* as the only natural host in the North Atlantic [37].

ISA viruses were first detected in wild trout and salmon at several locations in Scotland [38, 39] and later in the same two species collected in Norway [37, 40]. Based on analysis of the newly characterized surface protein, hemagglutinin-esterase [41, 42, 43, 44, 45], that contained a highly polymorphic region (HPR), a new hypothesis was launched, suggesting that wild salmonids could be an important reservoir for low-virulent ISA viruses (HPR0) in Norway [46]. It was further suggested that ISA emerges in farmed salmon after mutation in HPR0 variants transmitted from wild salmonids, followed by transmission between sites due to movement of salmon, well boat transport and other farming activities. It was speculated that the frequency of new primary outbreaks of ISA in farmed salmon could reflect natural variation in the prevalence of ISA virus in wild populations of salmonids. However, this view was dramatically changed in 2004/2005 when it was discovered that the HPR0 ISA viruses could be transmitted from farmed salmon brood fish via eggs, embryos, fry, parr and smolt, to postsmolt at marine sites [47, A. Nylund Pers. obs.]. The importance of vertical transmission of ISA viruses was further supported by a large study on transmission of ISA viruses based on results from of genotyping (molecular epizootics) and the observation of a high prevalence of HPR0 viruses in salmon at smolt production sites [48]. The study showed that ISA viruses from different areas along the Norwegian coast were closely related and that this relationship reflected the origin of the eggs from which the salmon were hatched. It was concluded that a limited number of ISA virus genotypes are circulating in the production cycle of farmed salmon and that there is little or no transmission of ISA viruses from wild to farmed salmonids. Several studies on genotyping of Norwegian ISA viruses based on segments five and six was published in the years that followed [37, 49, 50, 51, 52, 53], and the change from low-virulent HPR0 ISA viruses to high virulent isolates (HPRΔ) was suggested to be a result of inserts in front of the cutting site of the fusion protein in addition to changes in the HPR of segment six [46, 48, 49, 50, 54]. Presence of HPR0 viruses at smolt production sites and the importance of these viruses as a source for the virulent ISA virus strains (HPRΔ) was further supported by studies published in 2012 [53, 55]. These studies show that there are limited numbers of ISA viruses circulating in Norwegian salmon production. The mechanism of change from low-virulent to high virulent ISA viruses has later been addressed in several studies [53, 55, 56, 57, 58].

The uncontrolled high prevalence of HPR0 in farmed populations could pose a threat to wild populations of salmon, and wild salmon could also function as a vector for transmission of these low-virulent strains between farmed populations. Knowledge about the genome of ISA viruses in wild salmonids in Europe is absent with the exception of a short sequence of segment six from wild salmon in Scotland [39]. The aim of the present study was to provide new knowledge about ISA viruses in wild salmonids along the Norwegian coast, the possible impact of transmission of ISA viruses from farmed to wild salmonids, and wild salmonids as possible biological vectors for ISA viruses. To this end 21 sequences of ISAV segment 6 was obtained from wild salmon from Western, central and Northern Norway and phylogenetic analysis was conducted on these sequences and available segment 6 sequences from GenBank.

## Materials and methods

### Collection of wild salmonids

**Fig 1:**
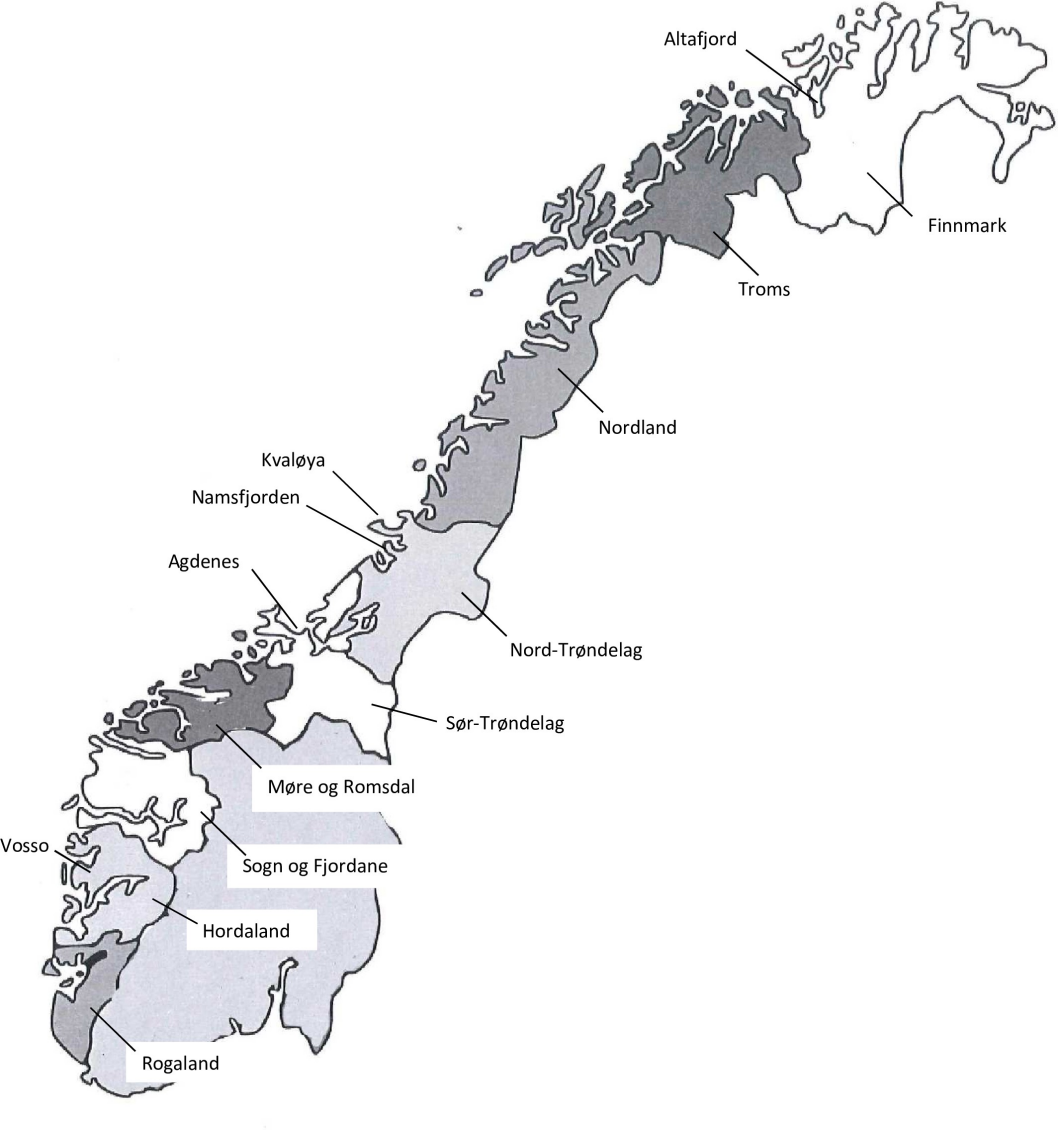
Overview of the major salmon farming counties in Norway; Rogaland–Finnmark. Wild salmonids (*Salmo salar* and *S*. *trutta*) were collected in rivers in western Norway (Rogaland–Møre og Romsdal), in the sea (Agdenes, Kvaløya, Namsfjorden) and rivers (Orkla, Gaula, Stjørdalselva, Steinkjærelva, Namsen) in Trøndelag, and in Altafjorden (sea) and Altaelva (river) in Finnmark.

Wild salmonids, *Salmo salar* and *Salmo trutta*, were collected from rivers in Western Norway, Trøndelag and Finnmark (Fig 1). All collected salmonids included in this study were killed following the Norwegian Animal Welfare Act (01.01.2010) and the regulations set by the Norwegian Food Safety Authority. The majority of the salmon (N = 436) from Western Norway were collected as brood fish from hatcheries for restocking of wild salmon in the period 2009–2013, while the trout (N = 253) were collected by electro-fishing in rivers and by net in the fjords. 60 salmon were collected by net in the inlet to river Vosso in 2011 (Fig 1). The salmon and trout from rivers in middle Norway (Trøndelag) and river Alta (Finnmark) were collected by fly-fishers, while the salmon and trout in the sea (Agdenes, Kvaløya, Namsfjorden and Altafjorden) were collected from fish traps (“kilenot”) (Fig 1 and Table 1). The tissue samples from all fish were sampled at the site after the fish had been killed according to the Norwegian standards, and the tissues were stored on ice or 70% ethanol during transportation to the Fish Diseases Research Group (FDRG) at the University of Bergen. Gills were sampled from all fish and, in addition, heart and kidney were taken from salmonids in Western Norway. The people responsible for the collection of salmonids and sampling of the tissues had permission form the local government, the Norwegian Environment Agency (project: 18S66D53 (808036)), and the river owners. The work on the tissues from the salmonids does not require approval from our institutional animal ethics committee.

**Table 1:** Overview of the prevalence of ISA virus positive wild salmonids sampled in rivers in Trøndelag (Orkla, Gaula, Stjørdalselva, Steinkjærelva, Namsen) and Finnmark (Altaelva). Salmon and trout were also collected in the sea using fish traps in the fjords; Agdenes, Kvaløya, Namsfjorden and Altafjorden (Fig 1). Gill tissues from all fish sampled were tested for presence of ISA virus.

	2013		2014		2015		2016		2017		2018		Total	
Species/Location	N	Pos—%	N	Pos—%	N	Pos—%	N	Pos—%	N	Pos—%	N	Pos—%	N	Pos—%
***Salmo salar***														
Agdenes	-	-	204	5–2.5	43	4–9.3	60	2–3.3	67	6–9.0	56	7–12.5	430	24–5.6
Orkla	70	4–5.7	70	2–2.9	72	4–5.6	28	0–0.0	27	0–0.0	-	-	267	10–3.7
Gaula	75	3–4.0	149	1–0.7	46	5–10.9	76	0–0.0	-	-	22	0–0.0	368	9–2.4
Stjørdalselva	74	2–2.7	16	2–12.5	50	0–0.0	12	0–0.0	60	3–5.0	78	2–2.6	290	9–3.1
Stenkjærelva	-	-	-	-	-	-	-	-	58	0–0.0	35	0–0.0	93	0–0.0
Kvaløya	-	-	-	-	-	-	-	-	-	-	60	6–10.0	60	6–10.0
Namsfjorden	-	-	-	-	94	20–21.3	62	4–6.5	58	22–**37.9**	60	18–30.0	274	64–**23.4**
Namsen	121	0–0.0	95	2–2.1	49	1–2.0	60	1–1.7	60	3–5.0	60	2–3.3	445	9–2.0
Altafjorden	-	-	-	-	-	-	-	-	41	2–4.9	38	8–21.1	79	10–12.7
Altaelva	-	-	-	-	-	-	80	5–6.3	81	4–4.9	60	3–5.0	221	12–5.2
***Salmo trutta***														
Agdenes	-	-	18	2–11.1	1	0–0.0	32	0–0.0	15	1–6.7	14	0–0.0	80	3–3.8
Kvaløya	-	-	-	-	-	-	-	-	-	-	8	1–12.5	8	1–12.5
Namsfjorden	-	-	-	-	2	1–50.0	1	0–0.0	2	0–0.0	7	0–0.0	12	1–8.3
Altafjorden	-	-	-	-	-	-	-	-	12	2–16.7	35	2–5.7	47	4–8.5

All RNA extractions, real time RT PCR, and sequencing of ISA viruses from positive tissues were carried out at the FDRG laboratory, University of Bergen.

### RNA extraction, PCR and sequencing

RNA was extracted using the protocol from Devold et al. [19]. In brief, Tissues were homogenised in 1 ml TRI-reagent (Sigma Aldrich, T9424) before the RNA was extracted once with 0.2 ml chloroform and precipitated in 0.5 ml isopropanol. Only a single isopropanol precipitation step was carried out.

Real time RT PCR was performed using the TaqMan Universal PCR Master Mix kit from Fisher Scientific (4304437) according to the manufacturers protocol and primers and probes from Plarre et al. [40] for ISAV segment 7 and with primer and probe targeting elongation factor α as a control [59]. ISAV positive samples with a CT-value below 30 were selected for sequencing.

First strand cDNA synthesis was carried out using the M-MLV kit (Promega, M170A and M531A). Approximately 2.0 μg of RNA and 1.0 pmol sequence-specific reverse transcription (RT) primer in a total volume of 10.0 μl was incubated at 70°C for 5 minutes and then immediately transferred to ice. RT reaction mix (1x M-MLV reaction buffer, 0.4 mM dNTPs and 100 U M-MLV) was added to each reaction to bring the final volume to 25.0 μl before the reaction was incubated at 37°C for one hour.

Four fragments covering the full length of segment six, were amplified by nested PCR using the Roche Expand HiFi PCR System (Roche, 4738276001) with one overlapping and one unique primer in the first and second reactions. In cases were a PCR product was obtained from one round of PCR, the product from the first PCR was used for sequencing. PCR fragments were sequenced in both directions using BigDye terminator 3.1 chemistry and the same primers as for PCR, resulting in overlapping sequence reads covering the full length of segment six in both directions.

### Phylogenetic analysis

The ISA virus segment six nucleotide sequences were assembled with the help of Vector NTI software (InforMax, Inc.). GenBank searches were done with blastn (2.7.1). The Vector NTI Suite software package (InforMax, Inc.) was used for the multiple alignments of the segment six sequences. To perform pairwise comparisons the multiple sequence alignment editor GeneDoc (Available at: www.psc.edu/biomed/genedoc) was used for manual adjustments. Sequences available from the EMBL nucleotide database were included in the comparisons (S1 Table).

The phylogenetic tree of all low-virulent ISAV (HPR0) was obtained by analysis of 1078 nt from segment six, while the phylogenetic trees, including both low–and high virulent ISA viruses, were obtained by analyses of 947 nucleotides from segment six (positions 61–1007 in the ORF, Accession no: AF302799). These trees were constructed using TREE-PUZZLE 5.2 (Available at: http://www.tree-puzzle.de), maximum likelihood (ML). The evolutionary model and substitution rates for the ML analyses were calculated for all datasets using jModelTest [60] with the Akaike Information Criterion option. The best model was GTR + I nucleotide evolution model with four category gamma rate. The maximum likelihood trees were bootstrapped (50000 puzzling steps) in TREE_PUZZLE. The HPR0 ISA virus from the USA (USA2004) was used as outgroup for the analysis of the low-virulent ISA viruses from Europe, while NT204a/15 and NT204b/15, from wild salmon in Norway, were used as out-group in the analysis including both low and high virulent viruses. Phylogenetic trees were drawn using TreeView [61].

#### Estimation of substitution rate and molecular dating

An alignment containing all available European ISAV HPR0 (N = 109) sequences covering the near full-length segment 6 (HE gene) was first imported into Mega6 [62] where a phylogenetic tree was obtained by Maximum likelihood under the General Time Reversible model [63] for nucleotide substitution, together with a discrete gamma model of rate heterogeneity amongst sites (GTR+G). Genetic distances in the obtained tree were further inspected in the software Tempest [64], with corresponding sequence sampling dates. The best fitting root for the tree was identified through regression, and a weak temporal structure (r2 = 0.08) was evident. Clade I contributed with a major part of the noise in the signal, and omitting this clade from the analysis resulted in a stronger correlation between genetic distance and sampling date (r2 = 0.26).

The alignment was then used for Bayesian phylogenetic analysis in BEAST 2 [65]. The HKY model of nucleotide substitution [66] was used with rates among sites modelled by a 4-category gamma distribution. The shape parameter of the gamma distribution and ratio of transition rate to transversion rate were estimated from the data. A relaxed uncorrelated clock model that allows substitution rate to vary between branches was employed. Rates were drawn from a lognormal distribution [67]. A flexible coalescent model allowing for change in population size (Bayesian Skyline Plot) was used as a prior to the tree [68]. Other priors were left as the default setting in beast. The Markov chain Monte Carlo algorithm was run for 115920000 generations and a burn-in of 10% was used. All effective sample size values were confirmed to be >500 when inspected in the software Tracer v1.7 [69].

A maximum clade credibility tree was made using TreeAnnotator v1.8.2, and visualized in Figtree v1.4. Geographical locations of nodes were inferred by parsimony, based on country origin of each sequence. Tips in the tree were assumed to provide equal amount of information on the probability of the shared node location. This probability was accumulated in deeper nodes. The calculated probability of each node location does therefore not take into account uncertainty in tree topology or the possibility of underlying patterns of geographical spread that are not captured by the information given in sample location. The tree was colored according to the most likely country where nodes have existed. Branches with less than 70% probability on location were not assigned to any particular country.

## Results

Low-virulent HPR0 ISA virus sequences have been obtained from all counties with commercial salmon farming in Norway, while ISA virus sequences from wild salmonids have only been obtained from the counties Rogaland, Sør Trøndelag, Nord Trøndelag and Finnmark (Fig 1). A total of 21 segment six sequences were obtained from wild salmonids in Norway (S1 Table). The prevalence of ISA virus in wild salmon (*Salmo salar*) and trout (*Salmo trutta*) in western Norway was low, 0.5% (2/436) and 1.6% (4/253), respectively. The densities of ISA virus RNA were also low in these samples and it was only possible to sequence segment six from one of the positives (R170/09), a trout collected in a river in Rogaland County (Fig 1 and S1 Table). This ISA virus had a deleted HPR (HPRΔ), but it is not known if this virus was virulent. The prevalence of ISA virus in salmonids collected in Nord- and Sør-Trøndelag, and Finnmark varied between the years of collection from 0.0% to 37.9% with the highest prevalence in wild salmon in Namsfjorden (Nord-Trøndelag) in 2017 (Fig 1 and Table 1). The prevalence of ISAV in the river Namsen that empties into Namsfjorden was only 5.0% the same year. Overall the prevalence’s of ISAV in the rivers are lower compared to that observed in the respective fjords (Table 1). The Ct values obtained for the elongation factor α, that was used as a control for the quality of the RNA extractions, ranged from 14.4 to 18.0 in the screening of the gill samples from the wild salmonids.

### Relationships between HPR0 ISA viruses

**Fig 2:**
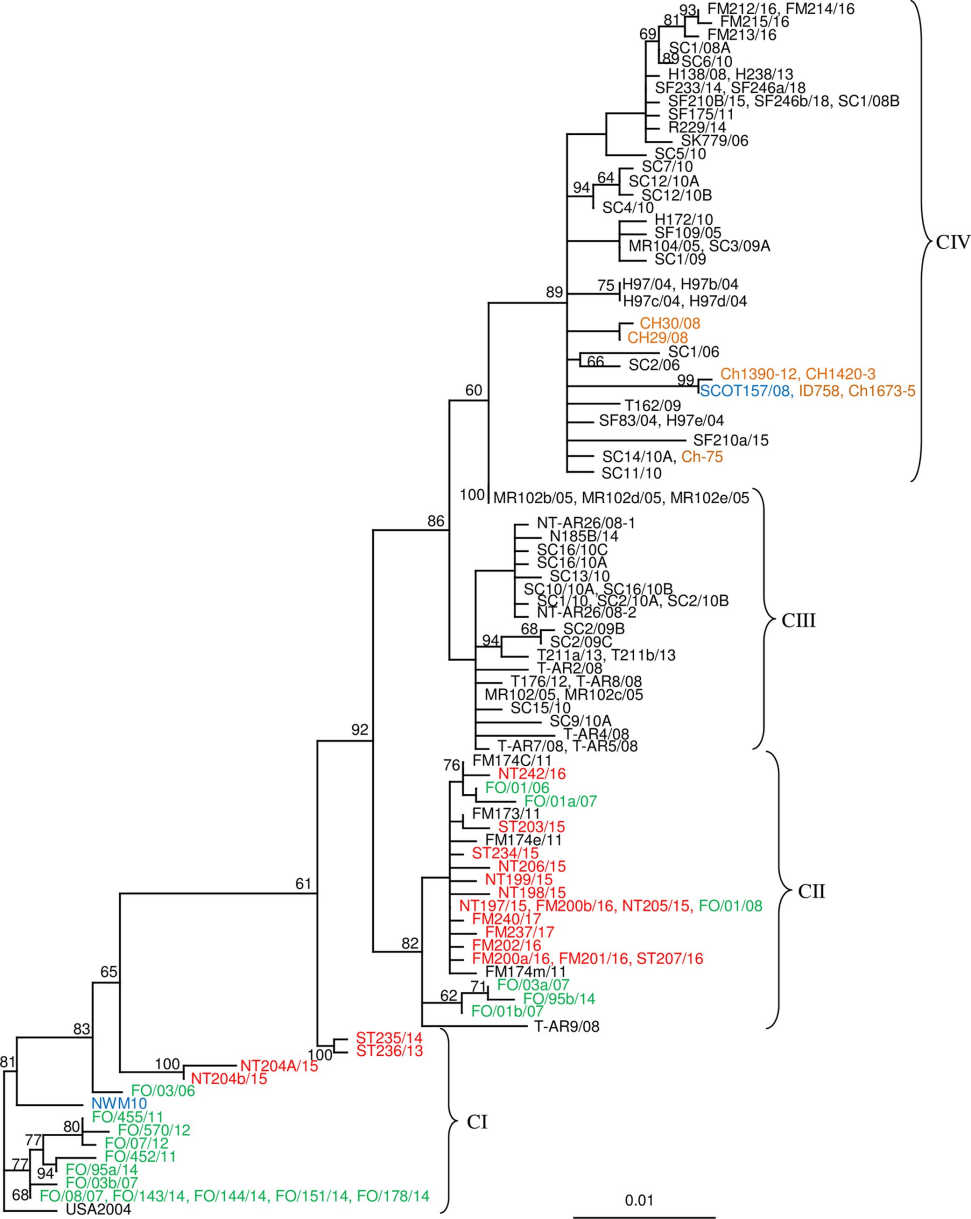
Phylogenetic relationship between all available HPR0 ISAV variants of European origin. These viruses group into four distinct clades (CI–CIV). The best-fitting nucleotide substitution model was used during maximum likelihood analysis and the tree was bootstrapped (50 000 quartet puzzling steps) in TREE_PUZZLE. The scale bar shows the number of nucleotide substitutions as a proportion of branch lengths. ISAV from farmed salmon in Norway (Black), Faeroe Islands (Green), Scotland (Blue), Chile (Orange), and wild salmonids in Norway (Red).

Phylogenetic analysis of segment six sequences (N = 118) from the low virulent (HPR0) ISA viruses grouped them into four major, supported, clades (C) named: CI, CII, CIII and CIV (Fig 2).

Clade I (CI) consists of viruses (N = 18) collected from farmed salmon in Faeroe Islands, Scotland, and USA, and from wild salmon collected in the sea in Trøndelag (ST235/14, ST236/13, NT204a/15, NT204b/15). These viruses were collected in the period 2004–2015. Members of this clade have not been detected in farmed salmon in Norway. The four HPR0 ISA viruses from wild salmon in Trøndelag constitute two separate subclades within CI.

Clade II (CII) consists of several HPR0 ISA viruses (N = 26) collected from wild and farmed salmon in Norway and farmed salmon in the Faeroe Islands collected in the period 2006–2018 (S1 Table and Fig 2). The ISA viruses from wild salmonids, collected in Trøndelag and Finnmark are closely related to HPR0 ISA viruses from farmed salmon in Troms, Finnmark and the Faeroe Islands. The HPR0 viruses from farmed Norwegian salmon in CII have been found in sea water only.

The ISAV (N = 28) in clade III (CIII), collected in the period 2005–2014, consists exclusively of low virulent ISA viruses from farmed Norwegian salmon (Fig 2). The viruses in CIII have been collected from production sites in both fresh and seawater at locations along the Norwegian coast, and include viruses from a brood fish location in fresh water (MR102/05, MR102b/05, MR102c/05, MR102d/05, MR102e/05).

The HPR0 ISA viruses (N = 46) in clade IV were collected in the period 2004–2018 from a large geographical area including Norway, Scotland and Chile (Fig 2). The viruses have been obtained from farmed salmon in both fresh and sea water. This clade also includes ISAV (H97/04, MR104/05, Scot157/08) from three different brood fish locations (fresh water), two in Norway and one in Scotland.

### Temporal structure and substitution rate

The relatively low correlation between genetic distance and sampling time (r2 = 0.08 with Clade I included and 0.26 without Clade I) suggested a high degree of rate variation between branches. A relaxed clock model where branches are allowed to evolve at different rates was therefore preferred for dating analyses. The BEAST analysis estimated the mean substitution rate to be 2.99 x 10^−4^ subst/site/yr, with a 95% Highest Posterior Density (HPD) interval of 1.84 to 4.21 x 10^−4^ subst/site/yr. The coefficient of variation was 0.66.

### BEAST analysis and molecular dating

**Fig 3:**
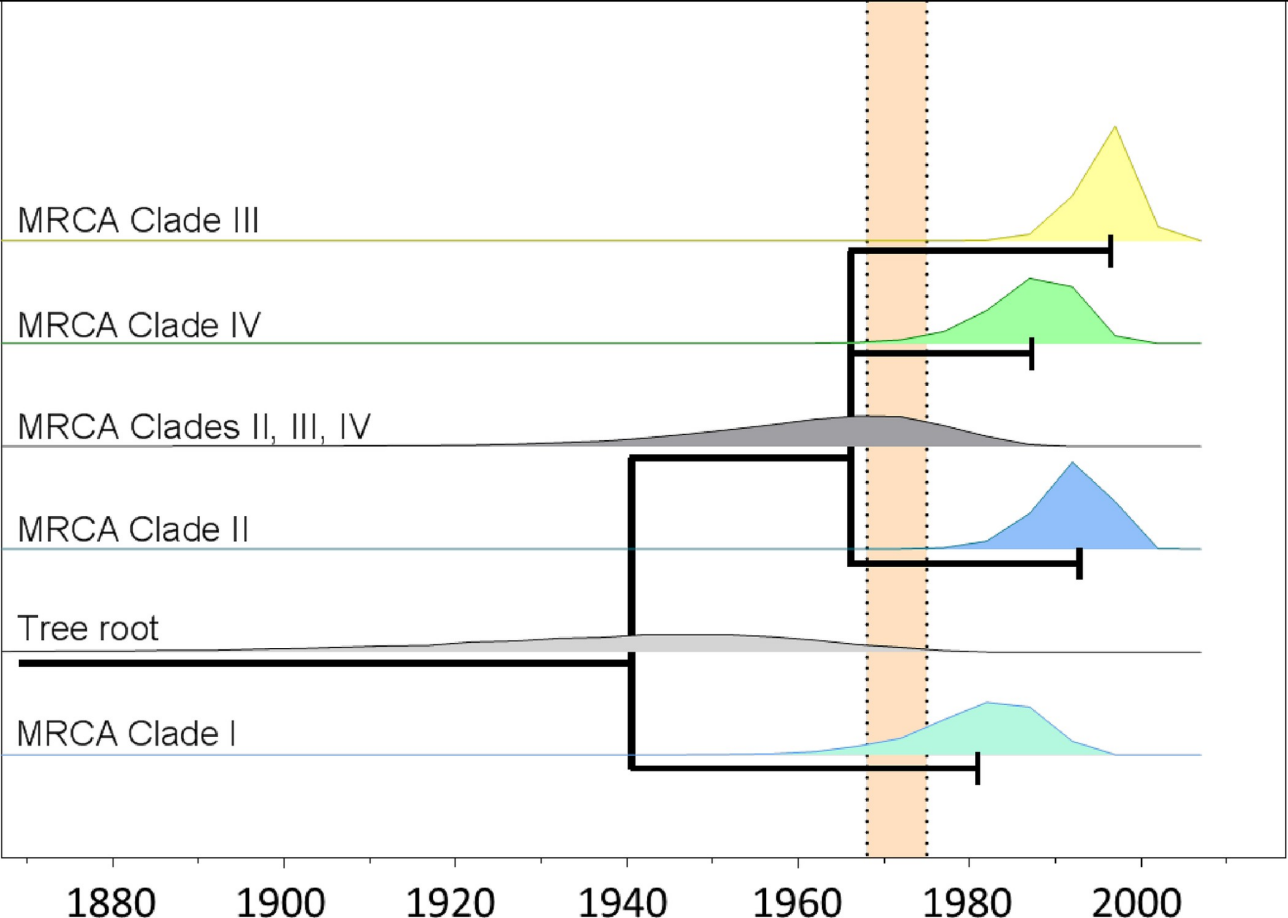
Highest posterior probability distributions for HPR0 MRCAs. Posterior probability distributions from major node ages in the BEAST-tree are shown with calendar year on the x-axis. Branch lengths are based on mean estimates for node ages, and tree tips represent the MRCAs of each of the four HPR0 clades. The time-period where domestication of Atlantic salmon began is illustrated with dotted lines.

The topology of the obtained BEAST tree confirmed the four general clades that had been identified in previous ML analyses, but the posterior probabilities of Clade III and several subnodes were low (S1 Fig). One sequence, T-AR9/08, did not conclusively locate to any of the clades. Divergence of Clade I from the other sequences was estimated to have occurred around the year 1941 (95% HPD interval: 1884–1976). The three other clades (II, III and IV) share a common ancestor that may have existed approximately around the time when modern salmonid farming began, although the estimate has a wide uncertainty interval (best estimate: 1966 with 95% HPD interval 1934–1987). All current sequence diversity within each of these three clades has likely evolved after the beginning of modern aquaculture in Europe, with estimates on node ages of 1997 (95% HPD interval: 1985–2004) for Clade III, 1987 (95% HPD interval: 1974–1997) for Clade IV and 1993 (95% HPD interval: 1977–2003) for Clade II (Fig 3).

### HPRΔ and HPR0

**Fig 4:**
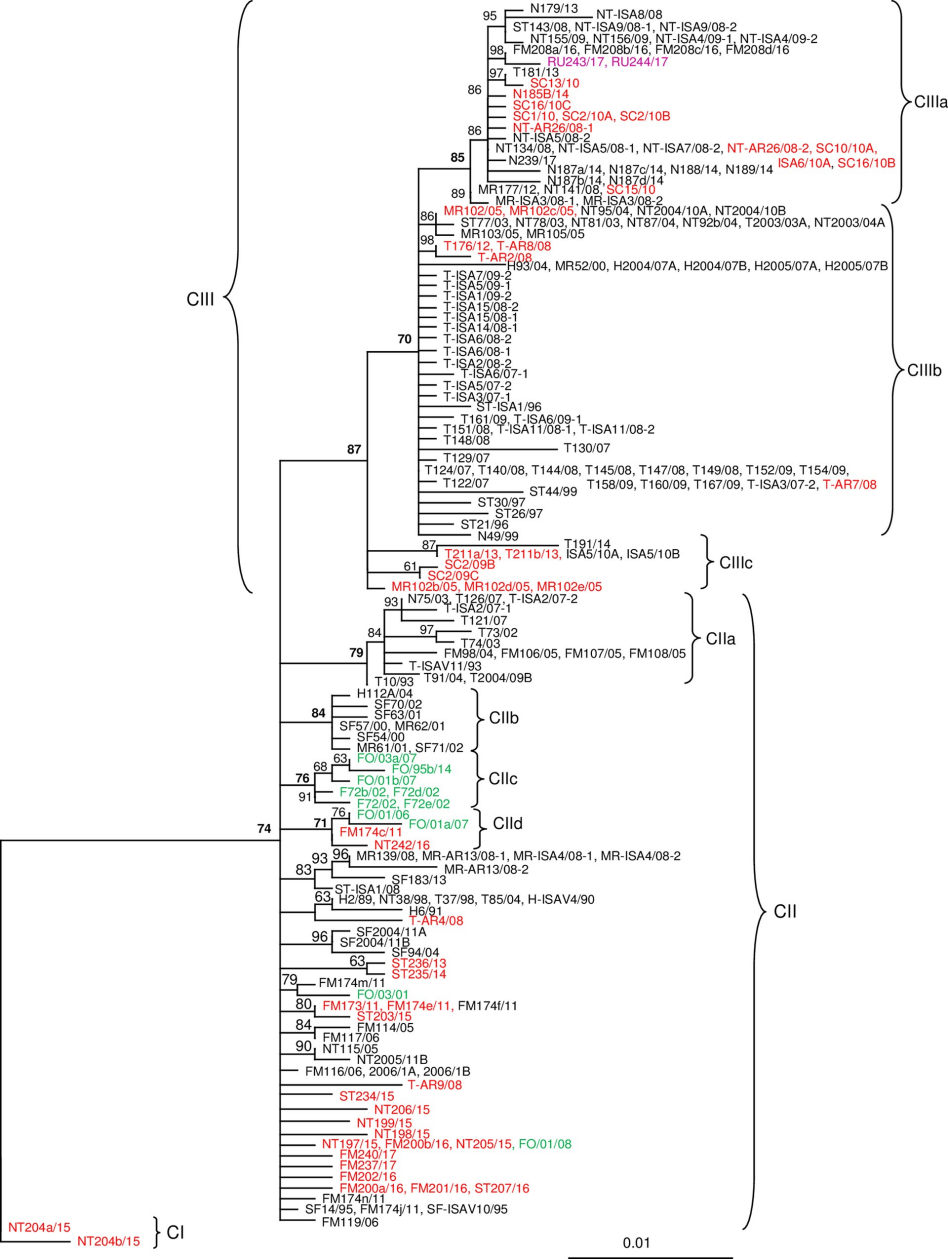
Relationships between European HPR0 and HPRΔ variants belonging to clades II and III. The best-fitting nucleotide substitution model was used during maximum likelihood analysis and the tree was bootstrapped (50 000 quartet puzzling steps) in TREE_PUZZLE. The scale bar shows the number of nucleotide substitutions as a proportion of branch lengths. HPRΔ ISAV from farmed salmon in Norway (Black), HPR0 ISAV from salmonids in Norway (Red), ISAV from the Faeroe Islands (Green), and ISAV from Russia (Purple).

**Fig 5:**
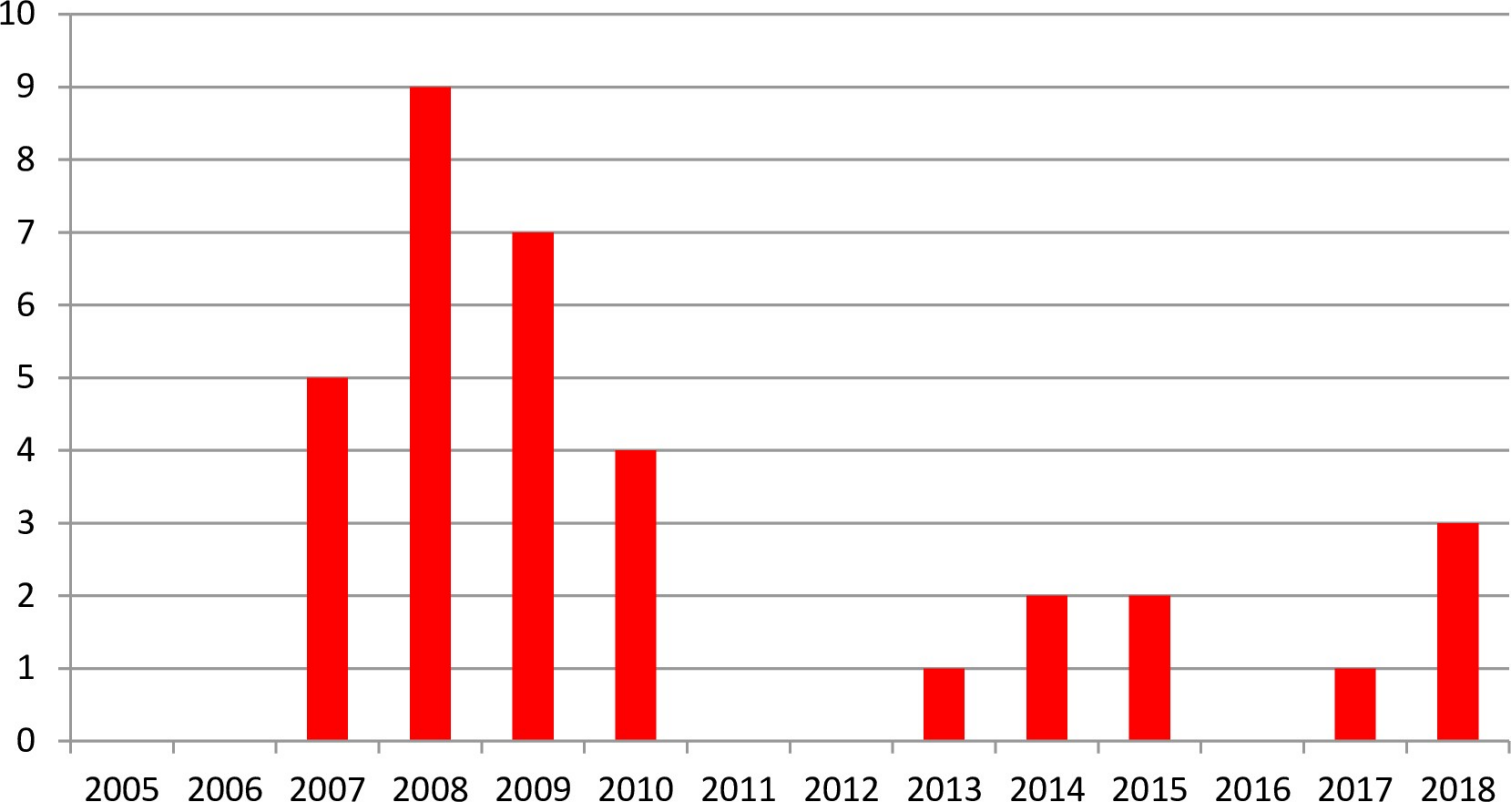
Number of ISA outbreaks in Troms County in the period 2005–2018.

Including virulent ISAV into the phylogenetic analyses shows that there are close relationships between the low virulent (HPR0) and high virulent (HPRΔ) viruses (Figs 4 and 5). The three major clades, CII–CIV, containing Norwegian ISAV are presented in more detail below.

Clade I contains only one known virulent ISAV (FO/121/14) from the Faroe Islands. This virus is identical to the HPR0 isolate (F0/07/12) from Faeroes based on analysis of the 947 nt from segment 6 (not included in Fig 2). A discussion of the emergence of FO/121/12 is presented by Christiansen et al. [58].

Clade II includes 75 ISA viruses from Norway and 11 from the Faeroe Islands (Fig 4). This clade contains 10 sub-clades with support values above 70.0%. One of the subclades, CIIa, contains 15 ISA viruses collected in the period 1993–2007. Alignment of the putative amino acid sequences shows that this group contains four HPRΔ variants, where the first is present in 1993 and 2003, the second in 2002, 2003, 2004 and 2005, the third in 2004, and the fourth in 2007. The first is present in both Troms (1993) and Nordland (2003), while the second is present in Troms and Finnmark. The distance in time and geographical occurrence suggests that the first HPRΔ variant, found in T10/93 and N75/03, represent two separate mutations from HPR0 variants which are also supported by the phylogenetic position of these within the subclade CIIa. All four variants are from the three northernmost counties in Norway.

Subclade CIIb contains eight viruses collected in the period 2000–2004 from salmon in three counties in western Norway (Fig 4). The seven viruses from 2000–2002 contain two different HPRΔ where the virus SF63/01 has a unique amino acid substitution that separates it from the other six. H112a/04 represents a completely different HPRΔ compared to the other members of CIIb.

Subclade CIIc contains both HPR0 and HPRΔ ISA viruses collected from salmon in the Faeroe Islands, while subclade CIId contains HPR0 ISA viruses from farmed salmon in Faeroe Islands and Finnmark county, and one HPR0 variant (NT242/16) from wild salmon in Trøndelag (Fig 4).

CII also contains one farmed HPR0 virus, FM174m/11, from Finnmark that groups together with a farmed HPR0 virus (FO/03/01) from the Faeroes, and three viruses from wild salmon (NT197/15, NT205/15, FM200b/16) from Northern Trøndelag and Finnmark that are identical to an HPR0 virus from the Faeroes (FO/01/08). Another result in CII worth mentioning is the four identical HPRΔ viruses (H2/89, NT38/98, T37/98, T85a/04) that were collected in three different counties in a period covering 16 years (Figs 1 and 4). These viruses could have arisen from the same HPR0 in four separate mutation events.

Clade III contains 115 ISA virus sequences from farmed salmon kept in fresh- and seawater in Norway and two viruses from farmed salmon kept at a marine site in Russia (Fig 4). The salmon in Russia originated from a Norwegian smolt production site.

Subclade, CIIIa, includes 43 ISA viruses collected in the period 2008–2017 from six different counties in Norway in addition to the two viruses from Russia (Fig 4). The sequences of SC16/10A and NT-ISA7/08-1 are not included in Fig 4 (S1 Table). CIIIa contains nine different HPRΔ ISA viruses in addition to HPR0 viruses. Five different HPRs, including HPR0, were present in the farmed salmon (N = 10) in 2008, while two HPRΔ and one HPR0 (N = 7) were present in 2014. One of the HPRΔ from 2008 emerged again in 2016 (eight years later) while another HPRΔ from 2008 emerged in 2013 (after five years). The distance in time and geographical occurrence suggests that they represent separate mutations from HPR0 variants. For seven ISA viruses in CIIIa, collected in 2008 and 2010, the part of the sequences of segment six (947 nt) used in the phylogenetic analysis are identical, but the viruses provide three different HPRs including HPR0. The two HPRΔ variants represent separate mutations. The three sequences NT141/08, SC15/10 and MR177/12 are also identical, but provide three different HPRs including HPR0. The virulent MR177/12 is from a brood fish location (S1 Table).

Subclade CIIIb contains 103 ISA viruses (HPR0 and seven different HPRΔ) collected in the period 1996–2012 where a high number of the viruses are from Troms County (Fig 4). 39 of these sequences (six from Nord Trøndelag, 31 from Troms and 2 with an unknown origin in Norway) are not included in Fig 3 due to lack of space (S1 Table). The viruses in this clade have been collected in six different counties (from Hordaland to Troms), and in the viruses collected in the period 1996–2005 six different HPR including HPR0 are present. These viruses represent, most likely, nine or more separate mutations from HPR0 viruses. CIIIb also includes two HPR0-viruses (MR102/05, MR102c/05) from a fresh water brood fish location and these are identical to two HPRΔ viruses NT95/04 and NT2004/10A from marine farms. With the exception of four low virulent (HPR0) virus (T-AR2/08, T-AR7/08, T-AR8/08, T176/12) and two other isolates (T144/08 and T152/09), the ISA viruses in CIIIb from Troms County collected in the period 2007–2012, all share the same HPRΔ. Salmon infected with ISAV escaped from a farm in this area early in the epizootic and this could have contributed to what seems to be a massive horizontal transmission of the virus resulting in a high number of outbreaks (Fig 5). However, including all ISA virus sequences (N = 76, S1 Table) collected from the 21 salmon production sites in Troms County that experienced ISA outbreaks in the period 2007–2009, provide seven different HPRΔ variants which show that, in addition to horizontal transmission, there is also a number of unique mutations suggesting several primary outbreaks in the area in this period. These additional ISAV from the outbreaks in Troms belong to other subclades (S1 Table).

Subclade CIIIc contains a few viruses (N = 10) collected in 2005–2014 from a fresh water brood fish location (MR102b/05, MR102d/05, MR102e/05) and from salmon at fresh- and sea water locations distant from the brood fish location (Fig 4). The remaining viruses, from marine farms, represented two different HPRΔ. The HPR0 sequences (947 nt) from the smolt in Troms (T211a/13 and T211b/13), collected in 2013, were identical to two HPRΔ viruses collected in 2010 (ISA5 10A and ISA5 10B).

**Fig 6:**
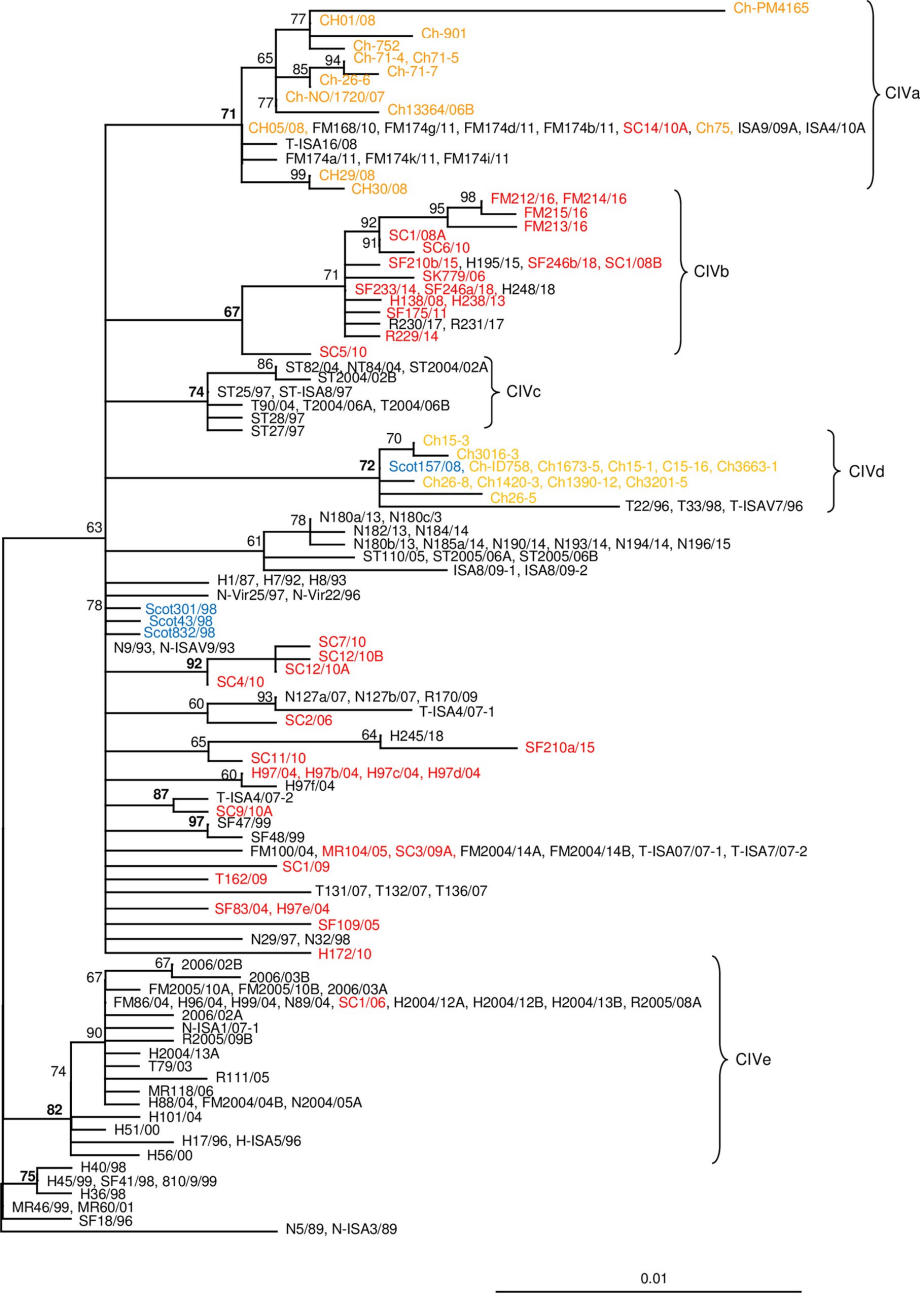
Relationships between European HPR0 and HPRΔ ISAV variants belonging to clade CIV. The best-fitting nucleotide substitution model was used during maximum likelihood analysis and the tree was bootstrapped (50 000 quartet puzzling steps) in TREE_PUZZLE. The scale bar shows the number of nucleotide substitutions as a proportion of branch lengths. HPRΔ ISAV from farmed salmon in Norway (Black), HPR0 ISAV from salmonids in Norway (Red), ISAV from Scotland (Blue), and ISAV from Chile (Orange).

The analysis of Clade CIV includes 224 ISA virus sequences collected from Norway, Scotland and Chile in the period from 1987–2018 (Fig 6). This clade also contains an ISA virus sequence (R170/09, HPRΔ) collected from wild trout (*Salmo trutta*) in 2009 in a river in Rogaland County (south western Norway). This sequence is identical to two ISA virus sequences, 947 nt (N127a/07 and N127b/07), collected from farmed salmon in Northern Norway (Nordland County) in 2007. Within CIV there are eight subclades with support values above 70.0%. The oldest ISAV sequence available (H1/87) belongs to this clade. 48 of the sequences from Chile, belonging to subclades CIVa and CIVd, are not included in Fig 5 due to lack of space (S1 Table).

Subclade CIVa is represented by 25 sequences from Norway and Chile, collected in the period 2006–2013, providing ten different HPRs including HPR0 sequences from both countries (Fig 6). Nine sequences in this subclade, collected from Norway and Chile in the period 2008–2011, are identical in the phylogenetic analysis (947 nt), but provide five different HPRs including HPR0 from both Chile (Ch-75) and Norway (SC14/10A). The ISA viruses detected during the first outbreaks of ISA in Chile in 2007 belong to this subclade.

Subclade CIVb (N = 21) is poorly supported (67.0%) and contains mainly HPR0 ISA virus sequences collected from Norwegian farmed salmon in the period 2006–2018 (Fig 6). The four HPRΔ sequences in this clade provide three different HPRs, one from two viruses in Rogaland (2017) and two others from two viruses (H195/15 and H248/18) from Hordaland. Four sequences (947 nt) are identical in the phylogenetic analyses and one of these, H195/15, is a HPRΔ variant from a brood fish location while the rest of the sequences are HPR0 sequences from fresh- and seawater production sites in Norway.

ISA viruses in subclade CIVc, collected in 1997 and 2004 in Trøndelag and Troms, consist exclusively of HPRΔ sequences (N = 11). The four viruses from 1997 (Trøndelag) have identical HPRΔ, while the viruses from 2004 provide two different HPRΔ where the two viruses from Troms have a different HPRΔ compared to those from Trøndelag and Nordland (Figs 1 and 6).

A group of ISA viruses, mainly from Chile and one virus from Scotland (collected in 2008–2013), constitute together with three viruses collected in Norway in 1996 and 1998, a distinct subclade, CIVd. This subclade includes 52 ISA virus sequences (S1 Table), providing four HPRs including HPR0, but only 16 of these sequences are presented in Fig 6. Two groups of ISA viruses in this subclade are identical (947 nt) in the phylogeny, and with sequences of both HPR0 and HPRΔ type. The three ISA viruses collected in Norway in 1996–1998 are distinctly different from the remaining ISA viruses collected 10 years later from Scotland and Chile. ISA virus Scot157/08 is from a brood fish location in Scotland.

Subclade CIVe consist of 29 ISA viruses collected in Norway in the period 1996–2007, and provides ten different HPRΔ and one HPR0. Nine sequences are identical in the phylogenetic analysis (947 nt), but provide six different HPRs including a HPR0. The HPRΔ sequences are from 2004, collected in Rogaland, Hordaland, Nordland and Finnmark, while the HPR0 sequence is from 2006 (Figs 1 and 6).

Among the remaining ISAV in CIV there are seven sequences from brood fish locations in fresh water, MR104/05 and H97/04—H97f/04. Five of the H97/04 sequences are HPR0 variants while H97f/04 is a HPRΔ variant. The HPRΔ variant was not associated with ISA.

## Discussion

This is the first study of European ISA viruses from farmed Atlantic salmon (*Salmo salar*) that includes the majority of available segment six sequences in addition to 21 ISA viruses from wild salmon (*Salmo salar*) and trout (*S*. *trutta*) in Norway. The study includes more than 100 low virulent HPR0 ISA viruses. The focus is on information (geographical occurrence, variants, transmission and reservoirs) that can be obtained from phylogenetic analysis of segment six from high (HPRΔ) and low (HPR0) virulent ISA viruses from fresh and sea water in Norway, Scotland, Faeroe Islands and Chile. This segment has been used in several earlier studies of the relationship between existing ISA viruses and for inferring short and long distance transmission [33, 34, 37, 41, 43, 46, 48, 49, 51, 52, 53, 55, 58, 70, 71, 72]. Admittedly, the change from low to high virulent variants of ISA viruses is, based on existing knowledge, dependent on mutational changes in both segment five (fusion protein, F, gene) and segment six (hemagglutinin-esterase, HE, gene) [49, 50, 53, 56, 57, 58], but all HPRΔ variants have so far been of higher virulence than HPR0 variants. Hence, in this study all HPRΔ variants are listed as virulent while the HPR0 variants are listed as low virulent viruses.

The prevalence of ISA viruses in wild salmonids varies between the different counties in Norway with a high registered prevalence in wild salmon in Namsfjorden and Altafjorden (Table 1). However, an even higher prevalence of ISAV was registered in trout (*S*. *trutta*) from rivers in Sogn og Fjordane County in 2001–2002 during outbreaks of ISA in salmon farms in the area [40]. The relatively high prevalence of ISAV in Namsfjorden and Altafjorden cannot be directly connected to outbreaks of ISA in these areas, but the prevalence of HPR0 ISAV variants can be high in farmed populations and may result in a spill over to wild salmon. However, there are no official systematic registrations of HPR0 ISAV in farmed salmon in Norway, meaning that the prevalence of ISAV in farmed populations in the study areas for the wild salmon can only be approximated based on the general knowledge of prevalence of this virus in farmed salmon [48, 53, 55]. The ISAV found in wild salmon in Trøndelag have not been seen in farmed salmon in this area giving little support to an assumed transmission between wild and farmed salmon, while nearly identical ISAV have been found in farmed and wild salmon in Finnmark (Fig 4). Hence, a recent transmission of ISAV between farmed and wild salmon may have occurred. The prevalence of ISAV in wild salmon in the sea is higher than the observed prevalence in neighbouring rivers to these marine collection sites (Table 1 and Fig 1). Hence, future studies should focus on the possible sublethal effects of HPR0 ISAV on salmon and if these low virulent viruses may influence on the survival of wild salmonids.

### Evolution of HPR0 ISAV

The analyzed ISAV HPR0 sequences showed a significant, but relatively noisy temporal signal. This suggests that the evolution of HPR0 viruses is clocklike only to a limited degree, and that branches in the tree evolve with variable rates on the time-scale that we have analyzed. This was accounted for in the BEAST analysis using a relaxed clock and a flexible coalescent model [67, 68] where branches are allowed to evolve with different rates, and population size can change over time. Comparing the time scale of evolutionary events with that of modern salmon farming, can inform on virus origin [73]. The four identified HPR0 clades do not share very recent ancestry. They are therefore not epidemiologically linked, and have probably evolved as separate lineages at least since the early days of industrialization of salmon farming in Europe (estimate for MRCA: 1966 (1934–1987)). Clade I, containing sequences from the Faroe Islands, Norway and the USA likely branched off in a period before modern farming where it appears less likely that human interaction was involved (although the relatively wide 95% HPD interval does stretch into the 1970s). The three remaining clades (Clade II, III, IV) all share a common ancestor that may have existed around the time when salmon farming emerged in Europe. This ancestor was the source of virus that since then evolved into 3 lineages that all contain recent isolates (2014 or younger). The lineages are therefore still ongoing and independent transmission chains.

HPR0 sequences from wild fish in Norway are limited to Clades I and II, where relatively recent jumps between farmed and wild hosts are indicated in Clade II. The direction of these jumps cannot be conclusively inferred from the tree. The two last clades (III and IV) contain HPR0 sequences from farmed fish only, and thus show no clear signs of interaction with wild salmonids. Although it can’t be excluded that wild fish infected with HPR0 ISAV belonging to Clades III and IV exist (undetected by us due to sampling bias), an alternative explanation to the lack of wild sources of viruses in these clades is that they are almost exclusively circulating in farmed populations. If that is the case, then transmission mechanisms are likely linked to industry specific operations (such as movement of fish or equipment), rather than passive transmission with water currents. The latter scenario would be expected to result in viruses from wild hosts distributed more equally to all clades. However, it should be added that an HPRΔ ISAV (R170/09) was detected in a wild trout sampled in a river in Rogaland County in 2009 and that identical viruses (N127a/07, N127b/07) had previously been detected in Nordland in 2007 (Fig 6). The connections between these two areas are not known, but a brood fish production site delivering embryos for smolt production to several locations in Norway, is located close to the river mouth in Rogaland.

### HPR0 ISAV

The HPR0 variants of ISA viruses have been known since 2002 and in 2003 it was suggested that the emergence of HPRΔ variants in farmed salmon resulted from deletions in the stalk region of the HE-gene [39, 46]. It is now commonly accepted that HPR0 variants are frequently present in farmed salmon in both fresh and sea water, including salmon brood fish, suggesting a large reservoir of the HPR0 viruses in farmed salmon [36, 46, 49, 53, 54, 55, 58, 74, 75, 76].

No ISAV from farmed salmon in Norway are present in clade, CI, however, this clade includes four viruses from wild salmon collected in Trøndelag. CI seems to represent a “mid-Atlantic” group of ISA viruses with a majority of the known viruses from the Faeroe Islands, but also with representatives from farmed salmon from Scotland and USA. The fact that members of this clade have not been found in farmed salmon in Norway, despite a relatively high prevalence of these viruses in wild salmon in Trøndelag, suggests that transmission from wild to farmed salmon is not occurring or is extremely limited. It is not known how this group of ISA viruses has been introduced into farmed salmon in the Faroes, but the limited genetic variation among these viruses, collected in the period 2006–2014, suggests a local reservoir in farmed salmon. HPR0 ISA viruses have been found in both fresh and seawater in the Faeroes [58]. The CI viruses from wild salmon in Norway (2013–2015) are only distantly related to those from farmed salmon in the Faeroes and give no indication of recent transmission between wild salmon and farmed salmon or *vice versa*.

The other three clades, CII–CIV, of HPR0 ISAV all include viruses from farmed salmon in Norway. However, only a few HPR0 members of CII have been found in farmed salmon in Norway, while the majority of the viruses from wild salmon in Norway and a number of ISA viruses from farmed salmon the Faeroes group in this clade. All HPR0 viruses in CII from farmed salmon are from Finnmark, the most Northern County in Norway (Figs 1 and 2). The CII-viruses from farmed salmon in Norway are closely related to viruses from wild salmon from both Finnmark and Trøndelag, and one virus, FO/01/08, from the Faeroes is identical to three viruses (NT197/15, NT205/15, FM200b/16) from wild salmon in Norway. The high identity between the wild and farmed ISA viruses in CII suggests recent transmissions. It cannot be excluded that wild salmon acts as a vector between farmed salmon in Norway and the Faeroes or, possibly less likely, that the wild salmon constitute a natural reservoir transmitting this group of ISA virus to farmed salmon. However, it is also known that salmon embryos from Norway have been moved to the Faroes and that the farmed salmon in this country is of Norwegian origin. Hence, it cannot be excluded that the members of CII, found in the Faeroes, could have been transmitted from Norway via movement of salmon embryos carrying HPR0 ISA viruses [34, 53], and that the presence of these viruses in wild salmon is a result of transmission from farmed salmon.

The HPR0 members of clade CIII (collected in the period 2005–2014) are all from farmed salmon in Norway including ISAV from a fresh water brood fish location, in addition to two viruses found in Russian salmon of Norwegian origin. This clade represents a group of viruses that seems to be maintained and circulated in populations of farmed Norwegian salmon with no indication of transmission to wild salmonids. The presence of HPR0 variants in both salmon brood fish and smolt in fresh water supports the hypothesis suggesting vertical transmission [34, 48, 53].

Clade CIV provides undisputable evidence of long distance transmission of HPR0 ISA viruses from Europe (Norway and Scotland) to Chile. This clade contains a number of HPR0 ISA viruses from Norway including viruses from two different brood fish locations in addition to a HPR0 variant from a Scottish brood fish population. Identical viruses to Scottish and Norwegians HPR0 viruses are present in Chile. This finding gives additional support to earlier studies presenting evidence on vertical transmission via embryos from Europe to Chile [34, 53]. Studies supporting vertical transmission of ISA viruses have also been published by others [77].

### HPR0 –HPRΔ ISA viruses

Norwegian salmon farming experienced a high number of ISA outbreaks in the period from 1989–1992 which resulted in the implementation of new management strategies such as one generation at each site only [3, 37]. In the period (1993–2018) after this new management there has been an average of 10 outbreaks each year ranging from one (1994 and 2011) to 23 (2000). This shows that the new management system, including the separation of generations, has prevented horizontal transmission of ISA virus from one generation to the next and, hence, lowered the infection pressure in the salmon farming areas. Still, the new management did not remove the ISA virus from farmed salmon in Norway. The discovery of a high prevalence of low virulent HPR0 ISAV in farmed salmon in both fresh and sea water, and the transition of these to high virulence HPRΔ variants, resulting from mutations in segments five and six, seems to be a major cause of new ISA outbreaks [46, 48, 49, 50, 53, 54, 55, 58]. This hypothesis is also strongly supported by the present study where HPR0 variants are found in all the major European ISA virus clades containing HPRΔ variants. Several cases where HPR0 and HPRΔ ISA viruses are identical in the phylogenetic analysis, based on 947 nt from the region coding the surface domain of the HE protein, suggest a recent transmission from low to high virulent variants (Figs 4 and 6). Six HE gene sequences (H97/04 –H97f/94) are presented from mature brood fish kept in fresh water showing the presence of two different HPR0 viruses and the presence of one HPRΔ variant (H97f/04) that differs from the HPR0 variants in one nucleotide only (Fig 6). It should be noted that ISA was not diagnosed in the brood fish population, and it is not known if the HPRΔ variant could cause disease in salmon. However, as already published, stress or maturing of salmon infected with HPR0 ISA virus seem to cause an increase in virus replication increasing the chances for mutations to occur [15, 16]. A possible transition from HPR0 to a virulent HPRΔ has also been documented in a recent publication from the Faroes [58]. Several HPR0 viruses from Norwegian brood fish are present in both clade CIII and CIV.

Since the discovery of vertical transmission of ISA virus via eggs from farmed salmon in 2005 there has been an ongoing discussion of the relative importance of vertical and horizontal transmission. The Chilean salmon farming industry will no doubt consider vertical transmission, after receiving both Norwegian and Scottish ISA virus via import of embryos, as very important [34, 53]; present study Fig 6). Considering the fact that the dominating ISA viruses in the Norwegian salmon farming industry belong to clade CIII and CIV, and not to the two clades where the majority known viruses from Norwegian wild salmon can be found, suggest that the viruses causing the majority of the ISA outbreaks in Norway are maintained or circulated in farmed salmon. It has already been thoroughly documented that HPR0 viruses can be found in brood fish, in fresh water production, and in salmon at marine sites [48, 53, 55]. Presence of ISA virus in brood fish and at fresh water sites, and the knowledge that the virus can be vertical transmitted, suggest that there could be a high frequency of transmission of HPR0 variants via brood fish in Norway. If this is an important transmission route for ISAV then one should expect to find different ISAV during the annual outbreaks of ISA in Norway. If, on the other hand, horizontal transmission is dominating the pattern should include locally (neighbouring farms) identical ISAV with identical HPRΔ. In Troms county in 2007–2009 the industry experienced 21 separate outbreaks of ISA where a number of these were closely related (CIIIb) and had the same HPRΔ. This was interpreted as an example of horizontal transmission resulting from a primary outbreak [52]. The analyses of our data give support this conclusion, but the situation was more complex, and the hypothesis suggesting an epizootic resulting from a primary outbreak does not give the complete picture of what happened in this area. A total of 78 HE sequences are available from the 21 outbreaks in Troms and these show the presence of seven unique HPRΔ variants which mean that 33.3% of the ISA viruses associated with disease in this area were not a result of horizontal transmission (S1 Table). This observation is best explained as a result of transition from HPR0 already present in the farmed salmon to virulent HPRΔ viruses [46, 48, 53, 55, 58].

Looking at the production period 2013–2018 the Norwegian salmon industry experienced ISA outbreaks in 70 farms and from these, 34 ISAV HPRΔ, representing 24 farms (34.3%) are included in the present study. These HPRΔ sequences provide 10 different HPR variants which means that they represent a minimum of 10 different new mutations events, ie. more than 41.7% of the viruses studied represent new mutations. It is not known if deleted HPRΔ may go through a new deletion, but if they all are deletions from HPR0 variants then close to 50% of the outbreaks could be related to primary outbreaks from HPR0 viruses. The HPR0 viruses that are related to these HPRΔ variants have only been found in farmed salmon and are most likely a result of a combination of vertical transmission and horizontal transmission within populations. It must also be added that viruses with similar HPRΔ have been found the same year as far apart as Sør-Trøndelag (ST77/03) and Troms (T2003/03A) counties suggesting separate mutation events and not horizontal transmission (Figs 1 and 6). When this occur the estimated percentage of new mutations resulting in primary outbreaks increases. Outbreaks of ISA in 15 farms a small area in Nordland county (2013–2017) included viruses presenting four different HPRΔ, ie. four separate mutation events. Again this indicates horizontal transmission, but with a significant contribution from independent mutation of HPR0 to HPRΔ variants.

### Conclusions

This study show that all known ISA viruses, HPR0 and HPRΔ, of European origin group into four major clades where members of three of these are present in Norwegian farmed salmon. One clade, CI, seems to have a mid-Atlantic distribution with the majority of the sequences obtained from farmed salmon in the Faeroe Islands. However, this group of viruses has also been detected in farmed salmon in Scotland, the USA and in a few wild salmon in Norway. The other three clades (CII, CIII and CIV) all contain ISAV from farmed salmon in Norway. CII consists of a collection of viruses from farmed salmon in the Faeroes and Norway and a number of ISAV from wild salmonids in Norway suggesting a recent transmission between these two countries and to wild salmon. CIII consists of ISAV exclusively from farmed salmon in Norway indicating circulation of this virus group within the salmon farming population, while CIV has a wider distribution being present in Norway, Scotland and Chile supporting the hypothesis of vertical transmission. A single HPRΔ ISAV from trout (*Salmo trutta*) from a Norwegian river is also present in CIV which could be a result of horizontal transmission between farmed and wild salmonids, however, this is a unique observation and the virus is not representative for the dominating ISAV in wild salmonids.

HPR0 variants are present in all clades and the existence of identical HPR0 and HPRΔ variants strongly supports the hypothesis suggesting a continuous generation of HPRΔ ISAV from the low virulent HPR0 variants. The high numbers of different HPRΔ variants found every year in Norway suggest that such transitions are of major importance for the yearly outbreaks of ISA in Norway. Even if ISAV from these primary outbreaks of ISA may transmit horizontal to neighbouring farms a targeted management will have to include a strong focus on the transmission of HPR0 variants of ISAV.

Based on these results we recommend that, due to a high occurrence of mutations of HPR0 variants resulting in HPRΔ viruses that cause outbreaks of ISA, a shift from focusing only on HPRΔ variants and outbreaks of ISA is needed. The industry should start focusing on removing the HPR0 ISAV from the brood fish core and the ISAV-free brood fish should be kept in closed containment systems (CCS) to prevent ISAV infection due to possible horizontal transmission from other farmed salmon. Even if we do not consider transmission of ISAV from wild salmonids as a major problem, the production of brood fish in CCS will prevent such transmission. Equally important is the protection of wild salmonids from virulent ISAV produced in farmed salmon. Presence of ISAV variants in farmed salmon, which are closely related to ISA viruses in wild salmonids, suggest that it cannot be excluded that wild salmonids are being infected by horizontal transmission from farmed salmon as is also seen between local salmon farms in close proximity to each other.

## Supporting information

S1 Table Overview of ISAV included in the study. Information about the ISA virus segment six sequences: clades (CI–CIV), HPR0 (Blue)- and HPRΔ (Black) variants, year of collection, sequence code, Accession numbers, sea water/fresh water, and host. NI in fig = not included in the figure. The ISAV are from all major salmon farming counties in Norway and from Scotland, Faeroe Islands, and Chile. A group of ISAV from Norway has been termed Unkown due to lack of information about county of origin and, for some, also the production site (fresh- or sea water) in published material [Kibenge et al. 2001; Markussen et al 2008; Aldrin et al. 2011; Lyngstad et al. 2011, 2012].
(PDF)

S1 Fig Dated phylogeny of HPR0 ISAV. The tree was obtained using BEAST with HKY+G model for nucleotide substitution and a relaxed uncorrelated clock model. Trees were constructed both under the assumption of a flexible (BSP) and a constant population size. Posterior probabilities are given for key nodes for both coalescent models (constant/BSP). Branches are scaled to calendar date, and colored according to most likely location based on parsimony. Branches with less than 70% probability on location are colored as uncertain.
(TIF)
