# Supplementary material for: Wild and farmed salmon (Salmo salar) as reservoirs for infectious salmon anaemia virus, and the importance of horizontal- and vertical transmission
Source: PLoS One. 2019 Apr 16;14(4):e0215478. doi: 10.1371/journal.pone.0215478 (PMC6467415; doi:10.1371/journal.pone.0215478)
Supplement: S1 Table — Information about the ISA virus segment six sequences: clades (CI–CIV), HPR0 (Blue)- and HPRΔ (Black) variants, year of collection, sequence code, Accession numbers, sea water/fresh water, and host. NI in fig = not included in the figure. The ISAV are from all major salmon farming counties in Norway and from Scotland, Faeroe Islands, and Chile. A group of ISAV from Norway has been termed Unkown due to lack of information about county of origin and, for some, also the production site (fresh- or sea water) in published material [Kibenge et al. 2001; Markussen et al 2008; Aldrin et al. 2011; Lyngstad et al. 2011, 2012]. (PDF) [file pone.0215478.s001.pdf]

S1 Table.

Deleted: Appendix

Norway

Rogaland

Clade	HPR	Year	Code	Accession		Host
CIVe	HPRΔ	2005	R111/05	DQ108609	SW	Farmed, *Salmo salar*
CIVe	HPRΔ	2005	R2005/09B	FM203273	SW	Farmed, *Salmo salar*
CIVe	HPRΔ	2005	R2005/08A	FM203276	SW	Farmed, *Salmo salar*
CIV	HPRΔ	2009	R170/09	MH286495	FW	Wild, *Salmo trutta*
CIVb	HPR0	2010	R229/14	MH397913	SW	Farmed, *Salmo salar*
CIVb	HPRΔ	2017	R230/17	MH397914	SW	Farmed, *Salmo salar*
CIVb	HPRΔ	2017	R231/17	MH397915	SW	Farmed, *Salmo salar*
**Hordaland**	**HPR**	**Year**	**Code**	**Accession**		**Host**
CIV	HPRΔ	1987	H1/87	AF364893	SW	Farmed, *Salmo salar*
CII	HPRΔ	1989	H2/89	AF220607	SW	Farmed, *Salmo salar*
CII	HPRΔ	1990	H-ISAV4/90	DQ785248	SW	Farmed, *Salmo salar*
CII	HPRΔ	1991	H6/91	AF364894	SW	Farmed, *Salmo salar*
CIV	HPRΔ	1992	H7/92	AF364898	SW	Farmed, *Salmo salar*
CIV	HPRΔ	1993	H8/93	AF309075	SW	Farmed, *Salmo salar*
CIVe	HPRΔ	1996	H17/96	AF364891	SW	Farmed, *Salmo salar*
CIVe	HPRΔ	1996	H-ISAV5/96	DQ785249	SW	Farmed, *Salmo salar*
CIV	HPRΔ	1998	H36/98	AF302799	SW	Farmed, *Salmo salar*
CII	HPRΔ	1998	H40/98	AF364877	SW	Farmed, *Salmo salar*
CIV	HPRΔ	1998	H45/99	AF364870	SW	Farmed, *Salmo salar*
CIVe	HPRΔ	2000	H51/00	AF364882	SW	Farmed, *Salmo salar*
CIVe	HPRΔ	2000	H56/00	AF364880	SW	Farmed, *Salmo salar*
CIVe	HPRΔ	2004	H88/04	AY973178	FW	Farmed, *Salmo salar*
CIIIb	HPRΔ	2004	H93/04	AY97379	SW	Farmed, *Salmo salar*
CIVe	HPRΔ	2004	H96/04	AY973180	FW	Brood fish, Salmo salar
CIV	HPR0	2004	H97/04	DQ108604	FW	Brood fish, *Salmo salar*
CIV	HPR0	2004	H97b/04	MH397901	FW	Brood fish, *Salmo salar*
CIV	HPR0	2004	H97c/04	MH397902	FW	Brood fish, *Salmo salar*
CIV	HPR0	2004	H97d/04	MH397903	FW	Brood fish, *Salmo salar*
CIV	HPR0	2004	H97e/04	MH397904	FW	Brood fish, *Salmo salar*
CVI	HPRΔ	2004	H97f/04	MH397905	FW	Brood fish, *Salmo salar*
CIVe	HPRΔ	2004	H99/04	AY973181	SW	Farmed, *Salmo salar*
CIVe	HPRΔ	2004	H101/04	DQ108602	SW	Farmed, *Salmo salar*
CIIb	HPRΔ	2004	H112A/04	DQ108603	FW	Farmed, *Salmo salar*
CIIIb	HPRΔ	2004	H2004/07A	FM203255	SW	Farmed, *Salmo salar*
CIIIb	HPRΔ	2004	H2004/07B	FM203256	SW	Farmed, *Salmo salar*
CIVe	HPRΔ	2004	H2004/12A	FM203264	SW	Farmed, *Salmo salar*
CIVe	HPRΔ	2004	H2004/12B	FM203265	SW	Farmed, *Salmo salar*
CIVe	HPRΔ	2004	H2004/13A	FM203266	SW	Farmed, *Salmo salar*
CIVe	HPRΔ	2004	H2004/13B	FM203267	SW	Farmed, *Salmo salar*
CIIIb	HPRΔ	2005	H2005/07A	FM203284	SW	Farmed, *Salmo salar*
CIIIb	HPRΔ	2005	H2005/07B	FM203285	SW	Farmed, *Salmo salar*
CIVe	HPRΔ	2006	H-2006/02A	FR751548	SW	Farmed, *Salmo salar*
CIVe	HPRΔ	2006	H-2006/02B	FR751549	SW	Farmed, *Salmo salar*

CIVb	HPR0	2008	H138/08	JN711063	FW	Farmed, *Salmo salar*
CIV	HPR0	2010	H172/10	JN711064	FW	Farmed, *Salmo salar*
CIVb	HPR0	2013	H238/13	MH397899	SW	Farmed, *Salmo salar*
CIVb	HPRΔ	2015	H195/15	MH397898	SW	Brood fish, *Salmo salar*
CIV	HPRΔ	2018	H245/18	MH397900	SW	Farmed, *Salmo salar*
CIVb	HPRΔ	2018	H248/18	MH986019	SW	Farmed, *Salmo salar*
**Sogn og Fjordane**	**HPR**	**Year**	**Code**	**Accession**		**Host**
CII	HPRΔ	1995	SF14/95	AF364873	SW	Farmed, *Salmo salar*
CII	HPRΔ	1995	SF-ISAV10/95	DQ785254	SW	Farmed, *Salmo salar*
CIV	HPRΔ	1996	SF18/96	AF364869	SW	Farmed, *Salmo salar*
CIV	HPRΔ	1998	SF41/98	AF364871	SW	Farmed, *Salmo salar*
CIV	HPRΔ	1999	SF48/99	AF364878	SW	Farmed, *Salmo salar*
CIV	HPRΔ	1999	SF47/99	AF364888	SW	Farmed, *Salmo salar*
CIIb	HPRΔ	2000	SF54/00	AF364884	SW	Farmed, *Salmo salar*
CIIb	HPRΔ	2000	SF57/00	AF364890	SW	Farmed, *Salmo salar*
CIIb	HPRΔ	2001	SF63/01	AY127879	SW	Farmed, *Salmo salar*
CIIb	HPRΔ	2002	SF70/02	AY127880	SW	Farmed, *Salmo salar*
CIIb	HPRΔ	2002	SF71/02	AY127881	SW	Farmed, *Salmo salar*
CIV	HPR0	2004	SF83/04	AY973190	FW	Farmed, *Salmo salar*
CII	HPRΔ	2004	SF94/04	DQ108611	SW	Farmed, *Salmo salar*
CII	HPRΔ	2004	SF2004/11A	FM203262	SW	Farmed, *Salmo salar*
CII	HPRΔ	2004	SF2004/11B	FM203263	SW	Farmed, *Salmo salar*
CIV	HPR0	2005	SF109/05	DQ108610	SW	Farmed, *Salmo salar*
CIVb	HPR0	2011	SF175/11	MH397918	SW	Farmed, *Salmo salar*
CII	HPRΔ	2013	SF183/13	MH397919	SW	Farmed, *Salmo salar*
CIVb	HPR0	2014	SF233/14	MH397922	SW	Farmed, *Salmo salar*
CIV	HPR0	2015	SF210a/15	MH397920	SW	Farmed, *Salmo salar*
CIVb	HPR0	2015	SF210b/15	MH397921	SW	Farmed, *Salmo salar*
CIVb	HPR0	2018	SF246a/18	MH986020	SW	Farmed, *Salmo salar*
CIVb	HPR0	2018	SF246b/18	MH986021	SW	Farmed, *Salmo salar*
CIIIa, NI in fig.	HPRΔ	2018	SF247/18	MH986022	SW	Farmed, *Salmo salar*
**Møre og Romsdal**	**HPR**	**Year**	**Code**	**Accession**		**Host**
CIV	HPRΔ	1999	MR46/99	AF364896	SW	Farmed, *Salmo salar*
CIIIb	HPRΔ	2000	MR52/00	AF364892	SW	Farmed, *Salmo salar*
CIV	HPRΔ	2001	MR60/01	AY127876	SW	Farmed, *Salmo salar*
CIIb	HPRΔ	2001	MR61/01	AY127877	SW	Farmed, *Salmo salar*
CIIb	HPRΔ	2001	MR62/01	AY127878	SW	Farmed, *Salmo salar*
CIIIb	HPR0	2005	MR102/05	DQ108605	FW	Brood fish location
CIIIc	HPR0	2005	MR102b/05	MH397906	FW	Brood fish location
CIIIb	HPR0	2005	MR102c/05	MH397907	FW	Brood fish location
CIIIc	HPR0	2005	MR102d/05	MH397908	FW	Brood fish location
CIIIc	HPR0	2005	MR102e/05	MH397909	FW	Brood fish location
CIIIb	HPRΔ	2005	MR103/05	DQ108606	SW	Brood fish, *Salmo salar*
CIV	HPR0	2005	MR104/05	DQ108607	FW	Brood fish location
CIIIb	HPRΔ	2005	MR105/05	DQ108608	SW	Brood fish, *Salmo salar*
CIVe	HPRΔ	2006	MR118/06	MH397910	SW	Farmed, *Salmo salar*
CII	HPRΔ	2008	MR139/08	JN711065	SW	Farmed, *Salmo salar*
CII	HPRΔ	2008	MR-AR13/08-1	FN687354	SW	Farmed, *Salmo salar*
CII	HPRΔ	2008	MR-AR13/08-2	FN687355	SW	Farmed, *Salmo salar*

CIIIa	HPRΔ	2008	MR-ISA3/08-1	FN687308	SW	Farmed, *Salmo salar*
CIIIa	HPRΔ	2008	MR-ISA3/08-2	FN687309	SW	Farmed, *Salmo salar*
CII	HPRΔ	2008	MR-ISA4/08-1	FN687310	SW	Farmed, *Salmo salar*
CII	HPRΔ	2008	MR-ISA4/08-2	FN687311	SW	Farmed, *Salmo salar*
CIIIa	HPRΔ	2012	MR177/12	MH397911	SW	Brood fish, *Salmo salar*
**Sør Trøndelag**	**HPR**	**Year**	**Code**	**Accession**		**Host**
CIIIb	HPRΔ	1996	ST21/96	AF364886	SW	Farmed, *Salmo salar*
CIIIb	HPRΔ	1996	ST-ISAV1/96	DQ785245	SW	Farmed, *Salmo salar*
CIIIb	HPRΔ	1997	ST26/97	AF364879	SW	Farmed, *Salmo salar*
CIVc	HPRΔ	1997	ST25/97	AF364885	SW	Farmed, *Salmo salar*
CIVc	HPRΔ	1997	ST27/97	AF364897	SW	Farmed, *Salmo salar*
CIVc	HPRΔ	1997	ST28/97	AF364875	SW	Farmed, *Salmo salar*
CIIIb	HPRΔ	1997	ST30/97	AY127875	SW	Farmed, *Salmo salar*
CIVc	HPRΔ	1997	ST-ISAV8/97	DQ785252	SW	Farmed, *Salmo salar*
CIIIb	HPRΔ	1999	ST44/99	AF302803	SW	Farmed, *Salmo salar*
CIIIb	HPRΔ	2003	ST77/03	AY973191	SW	Farmed, *Salmo salar*
CIVc	HPRΔ	2004	ST82/04	AY973192	SW	Farmed, *Salmo salar*
CIVc	HPRΔ	2004	ST2004/02A	FM203247	SW	Farmed, *Salmo salar*
CIVc	HPRΔ	2004	ST2004/02B	FM203248	SW	Farmed, *Salmo salar*
CIV	HPRΔ	2005	ST110/05	DQ108598	SW	Farmed, *Salmo salar*
CIV	HPRΔ	2005	ST2005/06A	FM203282	SW	Farmed, *Salmo salar*
CIV	HPRΔ	2005	ST2005/06B	FM203283		Farmed, *Salmo salar*
CIIIa	HPRΔ	2008	ST143/08	JN711073	SW	Farmed, *Salmo salar*
CII	HPRΔ	2008	ST-ISA1/08	FN687305	SW	Farmed, *Salmo salar*
CI	HPR0	2013	ST236/13	MH286494	FW	Wild, *Salmo salar*
CI	HPR0	2014	ST235/14	MH286493	FW	Wild, *Salmo salar*
CII	HPR0	2015	ST203/15	MH286490	SW	Wild, *Salmo salar*
CII	HPR0	2015	ST234/15	MH286492	FW	Wild, *Salmo salar*
CII	HPR0	2016	ST207/16	MH286491	SW	Wild, *Salmo salar*
**Nord Trøndelag**	**HPR**	**Year**	**Code**	**Accession**		**Host**
CII	HPRΔ	1998	NT38/99	AF364874	SW	Farmed, *Salmo salar*
CIIIb	HPRΔ	2003	NT78/03	AY973183	SW	Farmed, *Salmo salar*
CIIIb	HPRΔ	2003	NT81/03	AY973184	SW	Farmed, *Salmo salar*
CIIIb	HPRΔ	2003	NT2003/04A	FM203243	SW	Farmed, *Salmo salar*
CIIIb, NI in Figs	HPRΔ	2003	NT2003/06A	FM203245	SW	Farmed, *Salmo salar*
CIIIb, NI in Figs	HPRΔ	2003	NT2003/06B	FM203246	SW	Farmed, *Salmo salar*
CIIIb	HPRΔ	2004	NT2004/10A	FM203260	SW	Farmed, *Salmo salar*
CIIIb	HPRΔ	2004	NT2004/10B	FM203261	SW	Farmed, *Salmo salar*
CIIIb, NI in Figs	HPRΔ	2004	NT2004/03A	FM203290	SW	Farmed, *Salmo salar*
CIIIb, NI in Figs	HPRΔ	2004	NT2004/03B	FM203291	SW	Farmed, *Salmo salar*
CIIIb, NI in Figs	HPRΔ	2004	NT2004/08A	FM203257	SW	Farmed, *Salmo salar*
CIIIb, NI in Figs	HPRΔ	2004	NT2004/08B	FM203258	SW	Farmed, *Salmo salar*
CIVc	HPRΔ	2004	NT84/04	AY973185	SW	Farmed, *Salmo salar*
CIIIb	HPRΔ	2004	NT87/04	AY973186	SW	Farmed, *Salmo salar*
CIIIb	HPRΔ	2004	NT92B/04	AY973188	SW	Farmed, *Salmo salar*
CIIIb	HPRΔ	2004	NT95/04	AY973189	SW	Farmed, *Salmo salar*
CII	HPRΔ	2005	NT115/05	JN711068	SW	Farmed, *Salmo salar*
CII	HPRΔ	2005	NT2005/11B	FM203297	SW	Farmed, *Salmo salar*
CIIIa	HPRΔ	2008	NT134/08	JN711069	SW	Farmed, *Salmo salar*

CIIIa	HPRΔ	2008	NT141/08	JN711070	SW	Farmed, *Salmo salar*
CIIIa	HPR0	2008	NT-AR26/08-1	FN687356	SW	Farmed, *Salmo salar*
CIIIa	HPR0	2008	NT-AR26/08-2	FN687357	SW	Farmed, *Salmo salar*
CIIIa	HPRΔ	2008	NT-ISA5/08-1	FN687312	SW	Farmed, *Salmo salar*
CIIIa	HPRΔ	2008	NT-ISA5/08-2	FN687313	SW	Farmed, *Salmo salar*
CIIIa, NI in Figs	HPRΔ	2008	NT-ISA7/08-1	FN687316	SW	Farmed, *Salmo salar*
CIIIa	HPRΔ	2008	NT-SA7/08-2	FN687317	SW	Farmed, *Salmo salar*
CIIIa	HPRΔ	2008	NT-ISA8/08	FN687318	SW	Farmed, *Salmo salar*
CIIIa	HPRΔ	2008	NT-ISA9/08-1	FN687319	SW	Farmed, *Salmo salar*
CIIIa	HPRΔ	2008	NT-ISA9/08-2	FN687320	SW	Farmed, *Salmo salar*
CIIIa	HPRΔ	2009	NT155/09	JN711071	SW	Farmed, *Salmo salar*
CIIIa	HPRΔ	2009	NT156/09	JN711072	SW	Farmed, *Salmo salar*
CIIIa	HPRΔ	2009	NT-ISA4/09-1	FN687340	SW	Farmed, *Salmo salar*
CIIIa	HPRΔ	2009	NT-ISA4/09-2	FN687341	SW	Farmed, *Salmo salar*
CII	HPR0	2015	NT197/15	MH286482	SW	Wild, *Salmo salar*
CII	HPR0	2015	NT198/15	MH286483	SW	Wild, *Salmo salar*
CII	HPR0	2015	NT199/15	MH286484	SW	Wild, *Salmo salar*
CI	HPR0	2015	NT204a/15	MH286485	SW	Wild, *Salmo salar*
CI	HPR0	2015	NT204b/15	MH286486	SW	Wild, *Salmo salar*
CII	HPR0	2015	NT205/15	MH286487	SW	Wild, *Salmo salar*
CII	HPR0	2015	NT206/15	MH286488	SW	Wild, *Salmo salar*
**CIId**	HPR0	2016	NT242/16	MH286489	SW	Wild, *Salmo salar*
**CII, NI in Figs**	HPR0	2018	NT250/18	MK125504	SW	Wild, *Salmo salar*
**Nordland**	**HPR**	**Year**	**Code**	**Accession**		**Host**
CIV	HPRΔ	1989	N5/89	AY127882	SW	Farmed, *Salmo salar*
CIV	HPRΔ	1989	N-ISAV3/89	DQ785247	SW	Farmed, *Salmo salar*
CIV	HPRΔ	1993	N9/93	AF364895	SW	Farmed, *Salmo salar*
CIV	HPRΔ	1993	N-ISAV9/93	DQ785253	SW	Farmed, *Salmo salar*
CIV	HPRΔ	1996	N-Vir22/96	DQ785258	SW	Farmed, *Salmo salar*
CIV	HPRΔ	1997	N-Vir25/97	DQ785256	SW	Farmed, *Salmo salar*
CIV	HPRΔ	1997	N29/97	AF364872	SW	Farmed, *Salmo salar*
CIV	HPRΔ	1998	N32/98	AF364883	SW	Farmed, *Salmo salar*
CIIIb	HPRΔ	1999	N49/99	AF364876	SW	Farmed, *Salmo salar*
CIIa	HPRΔ	2003	N75/03	AY971661	SW	Farmed, *Salmo salar*
CIVe	HPRΔ	2004	N89/04	AY971662	SW	Farmed, *Salmo salar*
CIVe	HPRΔ	2004	N2004/05A	FM203251	SW	Farmed, *Salmo salar*
CIV	HPRΔ	2007	N127a/07	JN711066	SW	Farmed, *Salmo salar*
CIV	HPRΔ	2007	N127b/07	JN711067	SW	Farmed, *Salmo salar*
CIVe	HPRΔ	2007	N-ISA1/07-1	FN687284	SW	Farmed, *Salmo salar*
CIIIa	HPRΔ	2013	N179/13	MH397923	SW	Brood fish, *Salmo salar*
CIV	HPRΔ	2013	N180a/13	MH397924	SW	Farmed, *Salmo salar*
CIV	HPRΔ	2013	N180b/13	MH397925	SW	Farmed, *Salmo salar*
CIV	HPRΔ	2013	N180c/13	MH397926	SW	Farmed, *Salmo salar*
CIV	HPRΔ	2013	N182/13	MH397927	SW	Farmed, *Salmo salar*
CIV	HPRΔ	2014	N184/14	MH397928	SW	Farmed, *Salmo salar*
CIV	HPRΔ	2014	N185A/14	MH397929	SW	Farmed, *Salmo salar*
CIIIa	HPR0	2014	N185B/14	MH397930	SW	Farmed, *Salmo salar*
CIIIa	HPRΔ	2014	N187a/14	MH397931	SW	Farmed, *Salmo salar*
CIIIa	HPRΔ	2014	N187b/14	MH397932	SW	Farmed, *Salmo salar*

CIIIa	HPRΔ	2014	N187c/14	MH397933	SW	Farmed, *Salmo salar*
CIIIa	HPRΔ	2014	N187d/14	MH397934	SW	Farmed, *Salmo salar*
CIIIa	HPRΔ	2014	N188/14	MH397935	SW	Farmed, *Salmo salar*
CIIIa	HPRΔ	2014	N189/14	MH397936	SW	Farmed, *Salmo salar*
CIV	HPRΔ	2014	N190/14	MH397937	SW	Farmed, *Salmo salar*
CIV	HPRΔ	2014	N193/14	MH397938	SW	Farmed, *Salmo salar*
CIV	HPRΔ	2015	N194/15	MH397939	SW	Farmed, *Salmo salar*
CIV	HPRΔ	2015	N196/15	MH397940	SW	Farmed, *Salmo salar*
CIIIa	HPRΔ	2017	N239/17	MH397941	SW	Farmed, *Salmo salar*
**Troms**	**HPR**	**Year**	**Code**	**Accession**		**Host**
CIIa	HPRΔ	1993	T10/93	AF302801	SW	Farmed, *Salmo salar*
CIIa	HPRΔ	1993	T-ISAV11/93	DQ785255	SW	Farmed, *Salmo salar*
CIVd	HPRΔ	1996	T-ISAV7/96	DQ785251	SW	Farmed, *Salmo salar*
CIVd	HPRΔ	1996	T22/96	AF364889	SW	Farmed, *Salmo salar*
CIVd	HPRΔ	1998	T33/98	AF364887	SW	Farmed, *Salmo salar*
CII	HPRΔ	1998	T37/98	AF364881	SW	Farmed, *Salmo salar*
CIIa	HPRΔ	2002	T73/02	AY971663	SW	Farmed, *Salmo salar*
CIIa	HPRΔ	2003	T74/03	AY971664	SW	Farmed, *Salmo salar*
CIVe	HPRΔ	2003	T79/03	AY971665	SW	Farmed, *Salmo salar*
CIIIb	HPRΔ	2003	T2003/03A	FM203240	SW	Farmed, *Salmo salar*
CIIIb, NI in Fig.	HPRΔ	2003	T2003/03B	FM203241	SW	Farmed, *Salmo salar*
CIIIb, NI in Fig.	HPRΔ	2003	T2003/03C	FM203242	SW	Farmed, *Salmo salar*
CIIIb, NI in Fig.	HPRΔ	2003	T2003/06A	FM203245	SW	Farmed, *Salmo salar*
CIIIb, NI in Fig.	HPRΔ	2003	T2003/06B	FM203246	SW	Farmed, *Salmo salar*
CIIIb, NI in Fig.	HPRΔ	2004	T2004/08A	FM203257	SW	Farmed, *Salmo salar*
CIIIb, NI in Fig.	HPRΔ	2004	T2004/08A	FM203258	SW	Farmed, *Salmo salar*
CII	HPRΔ	2004	T85/04	AY971693	SW	Farmed, *Salmo salar*
CIVc	HPRΔ	2004	T90/04	AY971666	SW	Farmed, *Salmo salar*
CIIa	HPRΔ	2004	T91/04	AY971667	SW	Farmed, *Salmo salar*
CIVc	HPRΔ	2004	T2004/06A	FM203253	SW	Farmed, *Salmo salar*
CIVc	HPRΔ	2004	T2004/06B	FM203254	SW	Farmed, *Salmo salar*
CIIa	HPRΔ	2004	T2004/09B	FM203259	SW	Farmed, *Salmo salar*
CIIa	HPRΔ	2007	T121/07	JN711074	SW	Farmed, *Salmo salar*
CIIIb	HPRΔ	2007	T122/07	JN711075	SW	Farmed, *Salmo salar*
CIIIb	HPRΔ	2007	T124/07	JN711076	SW	Farmed, *Salmo salar*
CIIa	HPRΔ	2007	T126/07	JN711076	SW	Farmed, *Salmo salar*
CIIIb	HPRΔ	2007	T129/07	JN711077	SW	Farmed, *Salmo salar*
CIIIb	HPRΔ	2007	T130/07	MK125501	SW	Farmed, *Salmo salar*
CIV	HPRΔ	2007	T131/07	JN711078	SW	Farmed, *Salmo salar*
CIV	HPRΔ	2007	T132/07	MK125502	SW	Farmed, *Salmo salar*
CIV	HPRΔ	2007	T136/07	MK125503	SW	Farmed, *Salmo salar*
CIIa	HPRΔ	2007	T-ISA2/07-1	FN687286	SW	Farmed, *Salmo salar*
CIIa	HPRΔ	2007	T-ISA2/07-2	FN687287	SW	Farmed, *Salmo salar*
CIIIb	HPRΔ	2007	T-ISA3/07-1	FN687288	SW	Farmed, *Salmo salar*
CIIIb	HPRΔ	2007	T-ISA3/07-2	FN687289	SW	Farmed, *Salmo salar*
CIV	HPRΔ	2007	T-ISA4/07-1	FN687290	SW	Farmed, *Salmo salar*
CIV	HPRΔ	2007	T-ISA4/07-2	FN687291	SW	Farmed, *Salmo salar*
CIIIb, NI in Fig.	HPRΔ	2007	ISA5/07-1	FN687292	SW	Farmed, *Salmo salar*
CIIIb	HPRΔ	2007	T-ISA5/07-2	FN687293	SW	Farmed, *Salmo salar*

CIIIb	HPRΔ	2007	T-ISA6/07-1	FN687294	SW	Farmed, *Salmo salar*
CIIIb, NI in Fig.	HPRΔ	2007	T-ISA6/07-2	FN687295	SW	Farmed, *Salmo salar*
CIIIb, NI in Fig.	HPRΔ	2007	T-ISA6/07-3	FN687296	SW	Farmed, *Salmo salar*
CIIIb, NI in Fig.	HPRΔ	2007	T-ISA6/07-4	FN687297	SW	Farmed, *Salmo salar*
CIIIb, NI in Fig.	HPRΔ	2007	T-ISA6/07-5	FN687298	SW	Farmed, *Salmo salar*
CIIIb, NI in Fig.	HPRΔ	2007	T-ISA6/07-6	FN687299	SW	Farmed, *Salmo salar*
CIIIb, NI in Fig.	HPRΔ	2007	T-ISA6/07-7	FN687300	SW	Farmed, *Salmo salar*
CIIIb, NI in Fig.	HPRΔ	2007	T-ISA6/07-8	FN687301	SW	Farmed, *Salmo salar*
CIIIb, NI in Fig.	HPRΔ	2007	T-ISA6/07-9	FN687302	SW	Farmed, *Salmo salar*
CIV	HPRΔ	2007	T-ISA7/07-1	FN687303	SW	Farmed, *Salmo salar*
CIV	HPRΔ	2007	T-ISA7/07-2	FN687304	SW	Farmed, *Salmo salar*
CIIIb	HPRΔ	2008	T140/08	JN711079	SW	Farmed, *Salmo salar*
CIIIb	HPRΔ	2008	T144/08	JN711080	SW	Farmed, *Salmo salar*
CIIIb	HPRΔ	2008	T145/08	JN711081	SW	Farmed, *Salmo salar*
CIIIb	HPRΔ	2008	T147/08	JN711082	SW	Farmed, *Salmo salar*
CIIIb	HPRΔ	2008	T148/08	JN711083	SW	Farmed, *Salmo salar*
CIIIb	HPRΔ	2008	T149/08	JN711084	SW	Farmed, *Salmo salar*
CIIIb	HPRΔ	2008	T151/08	JN711085	SW	Farmed, *Salmo salar*
CIIIb, NI in Fig.	HPRΔ	2008	T-ISA2/08-1	FN687306	SW	Farmed, *Salmo salar*
CIIIb	HPRΔ	2008	T-ISA2/08-2	FN687307	SW	Farmed, *Salmo salar*
CIIIb	HPR0	2008	T-AR2/08	FN687348	SW	Farmed, *Salmo salar*
CIIIb	HPR0	2008	T-AR5/08	FN687350		Farmed, *Salmo salar*
CII	HPR0	2008	T-AR4/08	HE800144	FW	Farmed, *Salmo salar*
CIIIb	HPR0	2008	T-AR7/08	FN687351		Farmed, *Salmo salar*
CIIIb	HPR0	2008	T-AR8/08	FN687352		Farmed, *Salmo salar*
CII	HPR0	2008	T-AR9/08	FN687353		Farmed, *Salmo salar*
CIIIb	HPRΔ	2008	T-ISA6/08-1	FN687314	SW	Farmed, *Salmo salar*
CIIIb	HPRΔ	2008	T-ISA6/08-2	FN687315	SW	Farmed, *Salmo salar*
CIIIb, NI in Fig.	HPRΔ	2008	T-ISA10/08-1	FN687321	SW	Farmed, *Salmo salar*
CIIIb, NI in Fig.	HPRΔ	2008	T-ISA10/08-2	FN687322	SW	Farmed, *Salmo salar*
CIIIb	HPRΔ	2008	T-ISA11/08-1	FN687323	SW	Farmed, *Salmo salar*
CIIIb	HPRΔ	2008	T-ISA11/08-2	FN687324	SW	Farmed, *Salmo salar*
CIIIb, NI in Fig.	HPRΔ	2008	T-ISA12/08-1	FN687325	SW	Farmed, *Salmo salar*
CIIIb, NI in Fig.	HPRΔ	2008	T-ISA12/08-2	FN687326	SW	Farmed, *Salmo salar*
CIIIb, NI in Fig.	HPRΔ	2008	T-ISA13/08-1	FN687327	SW	Farmed, *Salmo salar*
CIIIb, NI in Fig.	HPRΔ	2008	T-ISA13/08-1	FN687328	SW	Farmed, *Salmo salar*
CIIIb	HPRΔ	2008	T-ISA14/08-1	FN687329	SW	Farmed, *Salmo salar*
CIIIb, NI in Fig.	HPRΔ	2008	T-ISA14/08-2	FN687330	SW	Farmed, *Salmo salar*
CIIIb	HPRΔ	2008	T-ISA15/08-1	FN687331	SW	Farmed, *Salmo salar*
CIIIb	HPRΔ	2008	T-ISA15/08-2	FN687332	SW	Farmed, *Salmo salar*
CIVa	HPRΔ	2008	T-ISA16/08	FN687333	SW	Farmed, *Salmo salar*
CIIIb	HPRΔ	2009	T152/09	JN711086	SW	Farmed, *Salmo salar*
CIIIb	HPRΔ	2009	T154/09	JN711087	SW	Farmed, *Salmo salar*
CIIIb	HPRΔ	2009	T158/09	JN711088	SW	Farmed, *Salmo salar*
CIIIb	HPRΔ	2009	T160/09	JN711089	SW	Farmed, *Salmo salar*
CIIIb	HPRΔ	2009	T161/09	JN711090	SW	Farmed, *Salmo salar*
CIV	HPR0	2009	T162/09	JN711091	FW	Farmed, *Salmo salar*
CIIIb, NI in Fig.	HPRΔ	2009	T-ISA1/09-1	FN687334	SW	Farmed, *Salmo salar*
CIIIb	HPRΔ	2009	T-ISA1/09-2	FN687335	SW	Farmed, *Salmo salar*

CIIIb, NI in Fig.	HPRΔ	2009	T-ISA2/09-1	FN687336	SW	Farmed, *Salmo salar*
CIIIb, NI in Fig.	HPRΔ	2009	T-ISA2/09-2	FN687337	SW	Farmed, *Salmo salar*
CIIIb, NI in Fig.	HPRΔ	2009	T-ISA3/09-1	FN687338	SW	Farmed, *Salmo salar*
CIIIb, NI in Fig.	HPRΔ	2009	T-ISA3/09-2	FN687339	SW	Farmed, *Salmo salar*
CIIIb	HPRΔ	2009	T-ISA5/09-1	FN687342	SW	Farmed, *Salmo salar*
CIIIb, NI in Fig.	HPRΔ	2009	T-ISA5/09-2	FN687343	SW	Farmed, *Salmo salar*
CIIIb	HPRΔ	2009	T-ISA6/09-1	FN687344	SW	Farmed, *Salmo salar*
CIIIb, NI in Fig.	HPRΔ	2009	T-ISA6/09-2	FN687345	SW	Farmed, *Salmo salar*
CIIIb, NI in Fig.	HPRΔ	2009	T-ISA7/09-1	FN687346	SW	Farmed, *Salmo salar*
CIIIb	HPRΔ	2009	T-ISA7/09-2	FN687347	SW	Farmed, *Salmo salar*
CIIIb	HPRΔ	2010	T167/10	JN711092	SW	Farmed, *Salmo salar*
CIIIb	HPR0	2012	T176/12	MH397942	SW	Farmed, *Salmo salar*
CIIIa	HPRΔ	2013	T181/13	MH397943	SW	Farmed, *Salmo salar*
CIIIc	HPR0	2013	T211a/13	MH397945	FW	Farmed, *Salmo salar*
CIIIc	HPR0	2013	T211b/13	MH397946	FW	Farmed, *Salmo salar*
CIIIc	HPRΔ	2014	T191/14	MH397944	SW	Farmed, *Salmo salar*

**Finnmark**	**HPR**	**Year**	**Code**	**Accession**		**Host**
CIVe	HPRΔ	2004	FM86/04	AY971659	SW	Farmed, *Salmo salar*
CIIa	HPRΔ	2004	FM98/04	AY971660	SW	Farmed, *Salmo salar*
CIV	HPRΔ	2004	FM100/04	AY971658	SW	Farmed, *Salmo salar*
CIVe	HPRΔ	2004	FM2004/04B	FM203250	SW	Farmed, *Salmo salar*
CIV	HPRΔ	2004	FM2004/14A	FM203268	SW	Farmed, *Salmo salar*
CIV	HPRΔ	2004	FM2004/14B	FM203269	SW	Farmed, *Salmo salar*
CIIa	HPRΔ	2005	FM106/05	DQ108599	SW	Farmed, *Salmo salar*
CIIa	HPRΔ	2005	FM107/05	DQ108600	SW	Farmed, *Salmo salar*
CIIa	HPRΔ	2005	FM108/05	DQ108601	SW	Farmed, *Salmo salar*
CII	HPRΔ	2005	FM114/05	JN711057	SW	Farmed, *Salmo salar*
CIVe	HPRΔ	2005	FM2005/10A	FM203288	SW	Farmed, *Salmo salar*
CIVe	HPRΔ	2005	FM2005/10B	FM203289	SW	Farmed, *Salmo salar*
CII	HPRΔ	2006	FM116/06	JN711058	SW	Farmed, *Salmo salar*
CII	HPRΔ	2006	FM117/06	MH397878	SW	Farmed, *Salmo salar*
CII	HPRΔ	2006	FM119/06	MH397879	SW	Farmed, *Salmo salar*
CIVa	HPRΔ	2010	FM168/10	JN711059	SW	Farmed, *Salmo salar*
CII	HPR0	2011	FM173/11	JN711060	SW	Farmed, *Salmo salar*
CIVa	HPRΔ	2011	FM174a/11	JN711061	SW	Farmed, *Salmo salar*
CIVa	HPRΔ	2011	FM174b/11	JN711062	SW	Farmed, *Salmo salar*
**CIId**	HPR0	2011	FM174c/11	MH397880	SW	Farmed, *Salmo salar*
CIVa	HPRΔ	2011	FM174d/11	MH397881	SW	Farmed, *Salmo salar*
CII	HPR0	2011	FM174e/11	MH397882	SW	Farmed, *Salmo salar*
CII	HPRΔ	2011	FM174f/11	MH397883	SW	Farmed, *Salmo salar*
CIVa	HPRΔ	2011	FM174g/11	MH397884	SW	Farmed, *Salmo salar*
CIVa	HPRΔ	2011	FM174i/11	MH397885	SW	Farmed, *Salmo salar*
CII	HPRΔ	2011	FM174j/11	MH397886	SW	Farmed, *Salmo salar*
CIVa	HPRΔ	2011	FM174k/11	MH397887	SW	Farmed, *Salmo salar*
CII	HPR0	2011	FM174m/11	MH397888	SW	Farmed, *Salmo salar*
CII	HPRΔ	2011	FM174n/11	MH397889	SW	Farmed, *Salmo salar*
CII	HPR0	2016	FM200a/16	MH286476	FW	Wild, *Salmo salar*
CII	HPR0	2016	FM200b/16	MH286477	FW	Wild, *Salmo salar*
CII	HPR0	2016	FM201/16	MH286478	FW	Wild, *Salmo salar*

CII	HPR0	2016	FM202/16	MH286479	FW	Wild, *Salmo salar*
CIIIa	HPRΔ	2016	FM208a/16	MH397890	SW	Farmed, *Salmo salar*
CIIIa	HPRΔ	2016	FM208b/16	MH397891	SW	Farmed, *Salmo salar*
CIIIa	HPRΔ	2016	FM208c/16	MH397892	SW	Farmed, *Salmo salar*
CIIIa	HPRΔ	2016	FM208d/16	MH397893	SW	Farmed, *Salmo salar*
CIVb	HPR0	2016	FM212/16	MH397894	FW	Farmed, *Salmo salar*
CIVb	HPR0	2016	FM213/16	MH397895	FW	Farmed, *Salmo salar*
CIVb	HPR0	2016	FM214/16	MH397896	FW	Farmed, *Salmo salar*
CIVb	HPR0	2016	FM215/16	MH397897	FW	Farmed, *Salmo salar*
CII	HPR0	2017	FM237/17	MH286480	FW	Wild, *Salmo salar*
CII	HPR0	2017	FM240/17	MH286481	SW	Wild, *Salmo salar*
**Unknown-Norway**	**HPR**	**Year**	**Code**	**Accession**		**Host**
CIV	HPRΔ	1999	810/9/99	AF378180	SW	Farmed, *Salmo salar*
CIVb	HPR0	**2006**	**SK779/06**	**EU118820**	SW	Farmed, *Salmo salar*
CII	HPRΔ	2006	2006/01A	FR751546	SW	Farmed, *Salmo salar*
CII	HPRΔ	2006	2006/01B	FR751547	SW	Farmed, *Salmo salar*
CIVe	HPRΔ	2006	2006/03A	FR751550	SW	Farmed, *Salmo salar*
CIVe	HPRΔ	2006	2006/03B	FR751551	SW	Farmed, *Salmo salar*
CIVe	HPR0	2006	SC1/06	HE800145		Farmed, *Salmo salar*
CIV	HPR0	2006	SC2/06	HE800146		Farmed, *Salmo salar*
CIII	HPR0	2008	AR2/08	FN687348	SW	Farmed, *Salmo salar*
CIIIb, NI in Fig.	HPR0	2008	AR3/08	FN687349	SW	Farmed, *Salmo salar*
CIVb	HPR0	2008	SC1/08A	HE800147		Farmed, *Salmo salar*
CIVb	HPR0	2008	SC1/08B	HE800148		Farmed, *Salmo salar*
CIV	HPRΔ	2009	ISA8-09-1	FR796471	SW	Farmed, *Salmo salar*
CIV	HPRΔ	2009	ISA8-09-2	FR796472	SW	Farmed, *Salmo salar*
CIV	HPR0	2009	SC1/09	HE800149		Farmed, *Salmo salar*
CIIIc	HPR0	2009	SC2/09B	HE800150		Farmed, *Salmo salar*
CIIIc	HPR0	2009	SC2/09C	HE800151		Farmed, *Salmo salar*
CIV	HPR0	2009	SC3/09A	HE800152		Farmed, *Salmo salar*
CIVa	HPRΔ	2009	ISA9/09A	HE800174	SW	Farmed, *Salmo salar*
CIIIa	HPR0	2010	SC1/10	HE800154		Farmed, *Salmo salar*
CIIIa	HPR0	2010	SC2/10A	HE800155		Farmed, *Salmo salar*
CIIIa	HPR0	2010	SC2/10B	HE800156		Farmed, *Salmo salar*
CIV	HPR0	2010	SC4/10	HE800157		Farmed, *Salmo salar*
CIVb	HPR0	2010	SC5/10	HE800158		Farmed, *Salmo salar*
CIVb	HPR0	2010	SC6/10	HE800159		Farmed, *Salmo salar*
CIV	HPR0	2010	SC7/10	HE800160		Farmed, *Salmo salar*
CIV	HPR0	2010	SC9/10A	HE800161		Farmed, *Salmo salar*
CIIIa	HPR0	2010	SC10/10A	HE800162		Farmed, *Salmo salar*
CIV	HPR0	2010	SC11/10	HE800163		Farmed, *Salmo salar*
CIV	HPR0	2010	SC12/10A	HE800164		Farmed, *Salmo salar*
CIV	HPR0	2010	SC12/10B	HE800165		Farmed, *Salmo salar*
CIIIa	HPR0	2010	SC13/10	HE800166		Farmed, *Salmo salar*
CIVa	HPR0	2010	SC14/10A	HE800167		Farmed, *Salmo salar*
CIIIa	HPR0	2010	SC15/10	HE800169		Farmed, *Salmo salar*
CIIIa, NI in Fig.	HPR0	2010	SC16/10A	HE800170		Farmed, *Salmo salar*
CIIIa	HPR0	2010	SC16/10B	HE800171		Farmed, *Salmo salar*
CIIIa	HPR0	2010	SC16/10C	HE800172		Farmed, *Salmo salar*

CIVa	HPRΔ	2010	ISA4/10A	HE800176	SW	Farmed, *Salmo salar*
CIIIc	HPRΔ	2010	ISA5/10A	HE800178	SW	Farmed, *Salmo salar*
CIIIc	HPRΔ	2010	ISA5/10B	HE800179	SW	Farmed, *Salmo salar*
CIIIa	HPRΔ	2010	ISA6/10A	HE800180	SW	Farmed, *Salmo salar*
**Scotland**	**HPR**	**Year**	**Code**	**Accession**		**Host**
CIV	HPRΔ	1998	Scot301/98	AF388581	SW	Farmed, *Salmo salar*
CIV	HPRΔ	1998	Scot43/98	AF302802	SW	Farmed, *Salmo salar*
CIV	HPRΔ	1998	Scot832/98	AF388582	SW	Farmed, *Salmo salar*
CI	HPR0	2007	NWM10	FJ178189	SW	Farmed, *Salmo salar*
**CIVd**	HPR0	2008	Scot157/08	JN711096	FW	Brood fish, *Salmo salar*
**Faeroe Islands**	**HPR**	**Year**	**Code**	**Accession**		**Host**
**CIIc**	HPRΔ	2002	F72/02	AF526263	SW	Farmed, *Salmo salar*
**CIIc**	HPRΔ	2002	F72b/02	AY971656	SW	Farmed, *Salmo salar*
**CIIc**	HPRΔ	2002	F72d/02	AY971657	SW	Farmed, *Salmo salar*
**CIIc**	HPRΔ	2002	F72e/02	DQ108612	SW	Farmed, *Salmo salar*
**CII**	HPRΔ	2001	FO/03/01	HQ664991	SW	Farmed, *Salmo salar*
**CIId**	HPR0	2006	FO/01/06	HQ664992	SW	Farmed, *Salmo salar*
**CI**	HPR0	2006	FO/03/06	HQ664993	SW	Farmed, *Salmo salar*
**CIIc**	HPR0	2007	FO/01b/07	HQ664995	SW	Farmed, *Salmo salar*
**CIIc**	HPR0	2007	FO/03a/07	HQ664996	SW	Farmed, *Salmo salar*
**CI**	HPR0	2007	FO/03b/07	HQ664997	SW	Farmed, *Salmo salar*
**CIId**	HPR0	2007	FO/01a/07	HQ664994	SW	Farmed, *Salmo salar*
**CI**	HPR0	2007	FO/08/07	HQ664998	SW	Farmed, *Salmo salar*
**CII**	HPR0	2008	FO/01/08	HQ664999	SW	Farmed, *Salmo salar*
**CI**	HPR0	2012	FO/07/12	KX823921	FW	Farmed, *Salmo salar*
**CI**	HPR0	2011	FO/452/11	KX823927	FW	Farmed, *Salmo salar*
**CI**	HPR0	2011	FO/455/11	KX823928	FW	Farmed, *Salmo salar*
**CI**	HPR0	2012	FO/570/12	KX823929	FW	Farmed, *Salmo salar*
CI, NI in Fig.	HPRΔ	2014	FO/121/14	KX823922	SW	Farmed, *Salmo salar*
**CI**	HPR0	2014	FO/95a/14	KX823930	SW	Farmed, *Salmo salar*
**CIIc**	HPR0	2014	FO/95b/14	KX823931	SW	Farmed, *Salmo salar*
**CI**	HPR0	2014	FO/143/14	KX823932	SW	Farmed, *Salmo salar*
**CI**	HPR0	2014	FO/144/14	KX823933	SW	Farmed, *Salmo salar*
**CI**	HPR0	2014	FO/151/14	KX823934	SW	Farmed, *Salmo salar*
**CI**	HPR0	2014	FO/178/14	KX823935	SW	Farmed, *Salmo salar*
**Russia**	**HPR**	**Year**	**Code**	**Accession**		**Host**
CIIIa	HPRΔ	2017	RU243/17	MH397916	SW	Farmed, *Salmo salar*
CIIIa	HPRΔ	2017	RU244/17	MH397917	SW	Farmed, *Salmo salar*
**USA**	**HPR**	**Year**	**Code**	**Accession**		**Host**
CI	HPR0	2004	USA2004	AY973194	SW	Farmed, *Salmo salar*
**Chile**						
CIVa	HPRΔ	2007	Ch-NO/1720/07	AM941715	SW	Farmed, *Salmo salar*
CIVa, NI in fig.	HPRΔ	2007	Ch26955-3	EU625679	SW	Farmed, *Salmo salar*
CIVa	HPRΔ	2008	Ch01/08	EU851043	SW	Farmed, *Salmo salar*
CIVa	HPRΔ	2008	Ch05/08	JN711093	SW	Farmed, *Salmo salar*
CIVa	HPR0	2008	CH29/08	JN711094	FW	Farmed, *Salmo salar*
CIVa	HPR0	2008	CH30/08	JN711095	FW	Farmed, *Salmo salar*
CIVa	HPRΔ	2008	Ch-PM4165	FJ594295	SW	Farmed, *Salmo salar*
CIVa	HPRΔ	2008	Ch13364/06B	FJ594284	SW	Farmed, *Salmo salar*

CIVa	HPR0		Ch-75	JQ712975		Farmed, *Salmo salar*
CIVa	HPRΔ	2009	Ch-752	GU830900	SW	Farmed, *Salmo salar*
CIVa	HPRΔ	2009	Ch-901	GU830908	SW	Farmed, *Salmo salar*
CIVa, NI in fig.	HPRΔ	2010	GIM-13968	MF314009		Farmed, *Salmo salar*
CIVa, NI in fig.	HPRΔ	2010	GIM-13552	MF314012		Farmed, *Salmo salar*
CIVa, NI in fig.	HPRΔ	2010	GIM-13970	MF314013		Farmed, *Salmo salar*
CIVa, NI in fig.	HPR0	2011	GIM-14046	MF314014		Farmed, *Salmo salar*
CIVa, NI in fig.	HPRΔ	2011	GIM-14332	MF314015		Farmed, *Salmo salar*
CIVa, NI in fig.	HPR0	2011	GIM-14542	MF314016		Farmed, *Salmo salar*
CIVd	HPR0	2012	Ch-ID758	KF019742		Farmed, *Salmo salar*
CIVd	HPRΔ	2013	Ch15-1	KF051855	SW	Farmed, *Salmo salar*
CIVd, NI in fig.	HPRΔ	2013	Ch15-2	KF051856		Farmed, *Salmo salar*
CIVd	HPRΔ	2013	Ch15-3	KF051857	SW	Farmed, *Salmo salar*
CIVd, NI in fig.	HPRΔ	2013	Ch15-4	KF051858		Farmed, *Salmo salar*
CIVd, NI in fig.	HPRΔ	2013	Ch15-5	KF051859	SW	Farmed, *Salmo salar*
CIVd	HPR0	2013	Ch1390-12	KF373253		Farmed, *Salmo salar*
CIVd	HPR0	2013	Ch1420-3	KF373255		Farmed, *Salmo salar*
CIVd, NI in fig.	HPRΔ	2013	Ch15-6	KF051860	SW	Farmed, *Salmo salar*
CIVd, NI in fig.	HPRΔ	2013	Ch15-7	KF051861		Farmed, *Salmo salar*
CIVd, NI in fig.	HPRΔ	2013	Ch15-8	KF051862	SW	Farmed, *Salmo salar*
CIVd, NI in fig.	HPRΔ	2013	Ch15-9	KF051863	SW	Farmed, *Salmo salar*
CIVd, NI in fig.	HPRΔ	2013	Ch15-10	KF051864	SW	Farmed, *Salmo salar*
CIVd, NI in fig.	HPRΔ	2013	Ch15-11	KF051865	SW	Farmed, *Salmo salar*
CIVd, NI in fig.	HPRΔ	2013	Ch15-12	KF051866	SW	Farmed, *Salmo salar*
CIVd, NI in fig.	HPRΔ	2013	Ch15-13	KF051867		Farmed, *Salmo salar*
CIVd, NI in fig.	HPRΔ	2013	Ch15-14	KF051868	SW	Farmed, *Salmo salar*
CIVd, NI in fig.	HPRΔ	2013	Ch15-15	KF051869	SW	Farmed, *Salmo salar*
CIVd	HPRΔ	2013	Ch15-16	KF051870	SW	Farmed, *Salmo salar*
CIVd	HPRΔ	2013	Ch26-5	KF051871	SW	Farmed, *Salmo salar*
CIVa	HPRΔ	2013	Ch26-6	KF051872	SW	Farmed, *Salmo salar*
CIVd	HPRΔ	2013	Ch26-8	KF051873	SW	Farmed, *Salmo salar*
CIVa	HPRΔ	2013	Ch71-4	KF051874	SW	Farmed, *Salmo salar*
CIVa	HPRΔ	2013	Ch71-5	KF051875		Farmed, *Salmo salar*
CIVa	HPRΔ	2013	Ch71-7	KF051876	SW	Farmed, *Salmo salar*
CIVa, NI in fig.	HPRΔ	2013	Ch71-8	KF051877		Farmed, *Salmo salar*
CIVa, NI in fig.	HPRΔ	2013	Ch71-9	KF051878		Farmed, *Salmo salar*
CIVd, NI in fig.	HPRΔ	2013	Ch77-16	KF051879		Farmed, *Salmo salar*
CIVd, NI in fig.	HPRΔ	2013	Ch77-17	KF051880		Farmed, *Salmo salar*
CIVd, NI in fig.	HPRΔ	2013	Ch77-18	KF051881	SW	Farmed, *Salmo salar*
CIVd, NI in fig.	HPRΔ	2013	Ch78-1	KF051882	SW	Farmed, *Salmo salar*
CIVd, NI in fig.	HPRΔ	2013	Ch78-2	KF051883		Farmed, *Salmo salar*
CIVd, NI in fig.	HPRΔ	2013	Ch78-3	KF051884	SW	Farmed, *Salmo salar*
CIVd, NI in fig.	HPRΔ	2013	Ch78-5	KF051885		Farmed, *Salmo salar*
CIVd, NI in fig.	HPRΔ	2013	Ch78-6	KF051886		Farmed, *Salmo salar*
CIVd, NI in fig.	HPRΔ	2013	CH78-7	KF051887		Farmed, *Salmo salar*
CIVd, NI in fig.	HPRΔ	2013	Ch78-8	KF051888	SW	Farmed, *Salmo salar*
CIVd, NI in fig.	HPRΔ	2013	Ch78-10	KF051889	SW	Farmed, *Salmo salar*
CIVd, NI in fig.	HPRΔ	2013	Ch78-11	KF051890		Farmed, *Salmo salar*
CIVd, NI in fig.	HPRΔ	2013	Ch78-12	KF051891		Farmed, *Salmo salar*

Formatted: Centered

Formatted: Centered

ClVd, NI in fig.	HPRΔ	2013	Ch78-14	KF051892		Farmed, *Salmo salar*
ClVd, NI in fig.	HPRΔ	2013	Ch78-15	KF051893		Farmed, *Salmo salar*
ClVd, NI in fig.	HPRΔ	2013	Ch78-16	KF051894	SW	Farmed, *Salmo salar*
ClVd, NI in fig.	HPRΔ	2013	Ch78-17	KF051895		Farmed, *Salmo salar*
ClVd, NI in fig.	HPRΔ	2013	Ch87-25	KF051896		Farmed, *Salmo salar*
ClVd, NI in fig.	HPRΔ	2013	Ch87-27	KF051897		Farmed, *Salmo salar*
ClVd, NI in fig.	HPRΔ	2013	Ch87-28	KF051898	SW	Farmed, *Salmo salar*
ClVd, NI in fig.	HPRΔ	2013	Ch87-30	KF051899	SW	Farmed, *Salmo salar*
ClVd, NI in fig.	HPRΔ	2013	Ch87-33	KF051900		Farmed, *Salmo salar*
ClVa, NI in fig.	HPRΔ	2013	GIM-HPR3a	MF314005		Farmed, *Salmo salar*
ClVd	HPRΔ	2013	Ch3201-5	KF373259	SW	Farmed, *Salmo salar*
ClVd	HPRΔ	2013	Ch3663-1	KF373260	SW	Farmed, *Salmo salar*
ClVd, NI in fig.	HPRΔ	2013	Ch3663-2	KF373261	SW	Farmed, *Salmo salar*
ClVd	HPRΔ	2013	Ch3016-3	KF386112	SW	Farmed, *Salmo salar*
ClVd	HPR0	2013	Ch1673-5	KF413751		Farmed, *Salmo salar*
ClVa, NI in fig.	HPR0	2015	GIM-19336	MF314010		Farmed, *Salmo salar*
ClVa, NI in fig.	HPRΔ	2015	GIM-19356	MF314036		Farmed, *Salmo salar*
