# Supplementary figures and images for: Wild and farmed salmon (Salmo salar) as reservoirs for infectious salmon anaemia virus, and the importance of horizontal- and vertical transmission
Source: PLoS One. 2019 Apr 16;14(4):e0215478. doi: 10.1371/journal.pone.0215478 (PMC6467415; doi:10.1371/journal.pone.0215478)

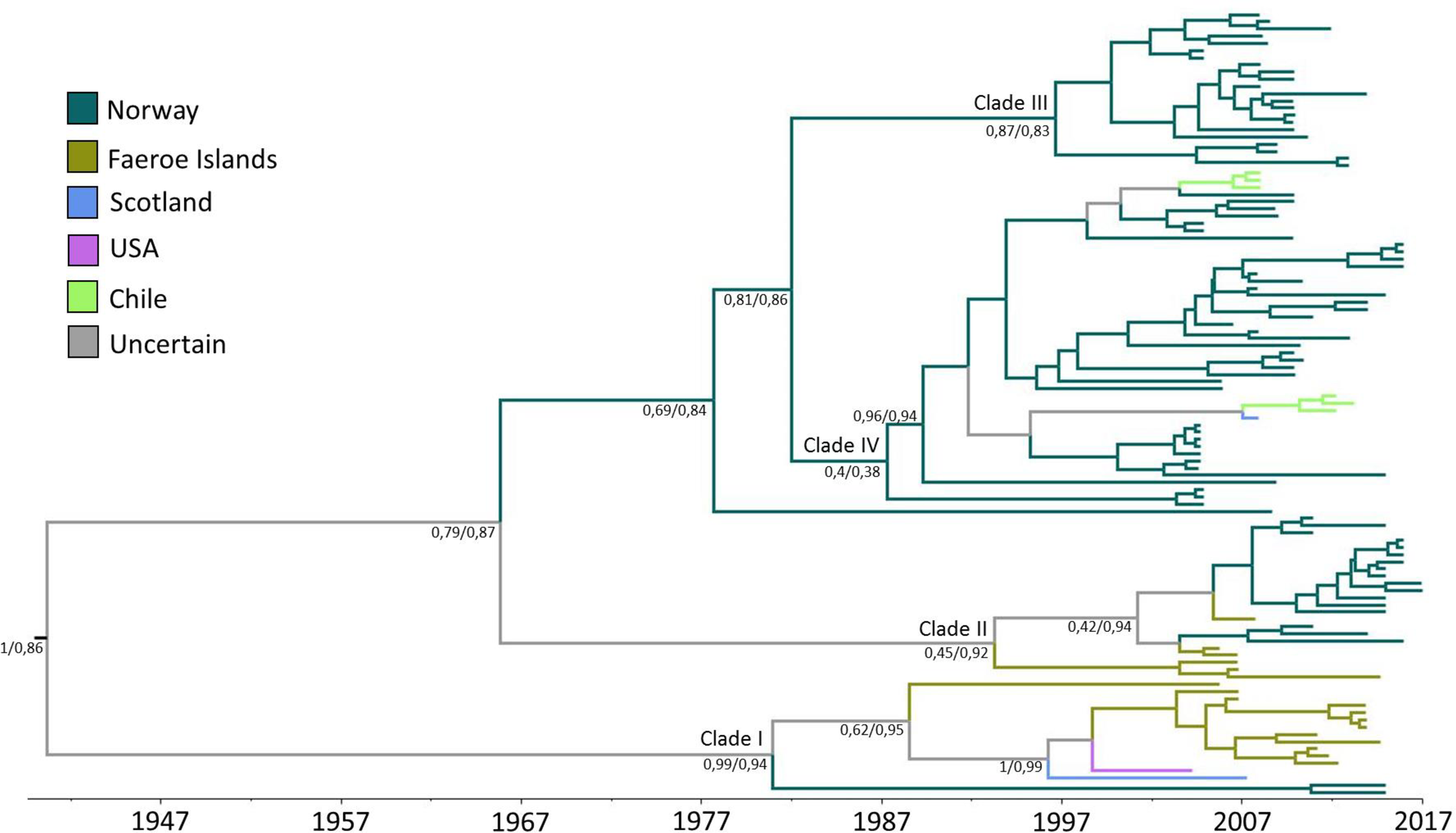

Supplement: S1 Fig — The tree was obtained using BEAST with HKY+G model for nucleotide substitution and a relaxed uncorrelated clock model. Trees were constructed both under the assumption of a flexible (BSP) and a constant population size. Posterior probabilities are given for key nodes for both coalescent models (constant/BSP). Branches are scaled to calendar date, and colored according to most likely location based on parsimony. Branches with less than 70% probability on location are colored as uncertain. (TIF) [file pone.0215478.s002.tif]
